# Absolute thermometry based on Brillouin scattering in gases

**DOI:** 10.1038/s41377-025-02168-3

**Published:** 2026-01-12

**Authors:** Yuting Yang, Marcelo A. Soto, Luc Thévenaz

**Affiliations:** 1 EPFL Ecole Polytechnique Fédérale de Lausanne, Institute of Electrical and Micro Engineering, Lausanne, Switzerland; 2 Department of Electronics Engineering, Universidad Técnica Federico Santa María, Valparaiso, Chile

**Keywords:** Optical physics, Optical sensors, Imaging and sensing, Optical metrology

## Abstract

We propose a novel thermometric technique for measuring absolute temperature in gas media based on Brillouin scattering. The method retrieves the temperature from the acoustic velocity of the gas, inferred through the spectral shift experienced by a scattered laser beam during the Brillouin acousto-optic interaction. This approach is inherently contactless, enabling remote sensing applications with high precision. It also exhibits enhanced sensitivity in the cryogenic range and is fully compatible with distributed measurements along recent hollow-core single-mode fibres. This study establishes the theoretical foundations of the technique and provides experimental validation across a range of temperature and pressure conditions. The influence of the gas species on the Brillouin response is analysed, enabling the selection of the optimal gas medium for specific applications. Illustrative distributed measurements demonstrate the strong potential of this technique for cryogenic sensing, where favourable scaling of several parameters leads to significantly improved temperature sensitivity. These results open new avenues for high-accuracy, remote, and minimally invasive thermometric measurements across a wide temperature range, including extreme cryogenic environments.

## Introduction

Temperature is one of the most frequently measured physical quantities, playing a crucial role in environmental monitoring and technical processes. While most applications rely on pre-calibrated contact probes, certain scenarios require minimal heat exchange with the measured object and demand absolute temperature readings. In such cases, contactless solutions like pyrometers^1^, which infer temperature from thermal radiation, are preferred. However, these techniques generally suffer from poor accuracy, especially at low temperatures where the emitted radiation is weak^2^. This highlights the pressing need for alternative methods enabling precise, non-contact, and absolute thermometry.

Optical fibres, cavities and waveguides offer an attractive platform for temperature sensing due to their immunity to chemical and electromagnetic interference, as well as their seamless integration into structures. Distributed fibre-optic sensing^3^ has gained wide adoption, particularly those based on Brillouin scattering^4^, where temperature information is extracted from the spectral position of the scattered light^5–7^. In solid-core silica fibres, this relies on the temperature-dependent acoustic velocity of the fibre material. However, accurate calibration is required to compensate for dopant effects and frozen-in strains^5–7^ and the temperature sensitivity diminishes sharply at cryogenic temperatures^8–10^, limiting the measurement precision in low-temperature environments.

Cryogenic temperature sensing presents a fundamental challenge for all fibre-based distributed sensors, not only those based on Brillouin scattering. Fibre Bragg grating (FBG) and Rayleigh-based sensors have also been used for cryogenic temperature sensing^11–13^, but their sensitivity and performance drop significantly at low temperatures, with special coatings only partially compensating for this limitation. On the other hand, Raman-based distributed sensors rely on thermally activated spontaneous scattering, producing very weak signals at cryogenic temperatures^14^. To enhance Raman signal detection and enable reliable measurements, photon-counting detection has been shown beneficial^14^.

To overcome the limitations of optical fibre sensing at cryogenic temperatures, an alternative approach is to replace the fibre with an optical cavity. Cryogenic thermometry has been demonstrated using cavities filled with liquid helium, which have been successfully employed to measure Brillouin scattering at deep cryogenic temperatures^15^. Furthermore, highly sensitive thermometry, though not at cryogenic temperatures, has been achieved using Doppler velocimetry of light-propelled microparticles in hollow-core fibres, reaching an exceptional sensitivity of approximately 97 MHz/K and a spatial resolution of 5 mm^16,17^.

In this work, we introduce a novel method for absolute, contactless thermometry based on Brillouin scattering in gases. Unlike in solids, where waveguide and material characteristics complicate interpretation, the Brillouin spectral shift in gases directly reflects the thermodynamic temperature, with minimal structural dependence. We demonstrate this principle using a hermetically sealed hollow-core optical fibre (HCF) filled with gases, achieving highly accurate and unambiguous temperature readings. Unlike silica-based fibre sensors, which generally require calibration and provide only relative or differential measurements, the method proposed here enables absolute thermometry. This is achieved through the full predictability of the Brillouin response using gas state equations, allowing temperature to be measured directly without the need for calibration or external references. Interestingly, the sensitivity increases at low temperatures, making this approach particularly well-suited for cryogenic applications. Beyond fibre-based implementations, this method can be extended to free-space configurations such as Brillouin microscopy^18,19^, enabling precise, absolute temperature measurements of gases without physical contact. This work not only identifies optimal gas candidates for sensing but also establishes a versatile framework for distributed and localised thermometry in environments where conventional techniques fall short.

## Results

### Stimulated Brillouin scattering in gases

Backward stimulated Brillouin scattering (SBS)^4,20^ is a nonlinear, inelastic optical process where two counterpropagating optical waves—the pump and the probe—interact in resonance with an acoustic wave generated through electrostriction and optical interference. This resonance occurs when the Bragg condition is satisfied, leading to energy transfer from a higher-frequency pump to a lower-frequency probe. To satisfy the Bragg condition, the acoustic wave frequency ${{\nu }}_{B}$ must be a fraction of the optical pump frequency ${{\nu }}_{p}$, scaled by the ratio of the acoustic velocity ${V}_{a}$ to the optical phase velocity ${V}_{p}$ in the medium. Expressing this relation in a more practical form using the effective refractive index ${n}_{{eff}}$ and the pump wavelength ${{\lambda }}_{0}$ in vacuum, the acoustic wave frequency ${{\nu }}_{B}$ can be written as^4^:

1 $${{\rm{\nu }}}_{B}=2\frac{{V}_{a}}{{V}_{p}}{{\rm{\nu }}}_{p}={2n}_{{eff}}\frac{{V}_{a}}{{{\rm{\lambda }}}_{0}}$$

Brillouin scattering modulates the refractive index of the medium (solid, liquid, or gas), with a maximum interaction occurring when the frequency difference between the pump and probe matches the acoustic wave frequency ${{\nu }}_{B}$, defining the Brillouin frequency shift (BFS) of the medium^4^. This BFS provides a direct measurement of the acoustic velocity, which varies with environmental factors, like temperature or strain^5–7^.

For a pump light in the near infrared, the backward BFS spans from a few hundred of MHz in gases^21^, to tens of GHz in solids^22–24^, with resonance spectral widths from sub-kHz to a few MHz. It should be also mentioned that other scheme, such as guided wave acoustic Brillouin scattering^25^, and mechanically structured fibres such as dual-web fibres^26^, show BFS in the MHz range, but are based on a distinct phase-matching scheme. By monitoring the BFS, changes in the acoustic velocity can be precisely tracked. This property is widely utilised in standard silica fibres for sensing applications, particularly for temperature and strain measurements^5–7^. However, silica fibres require a precise BFS calibration because their acoustic velocity is affected by dopants shaping the refractive index profile and by frozen residual strains created from the rapid cooling during the drawing process. In addition, its temperature dependence is known only empirically and becomes non-monotonic at low temperatures, where variations with temperature eventually vanish^8–10^.

In contrast, purified gases consist of a single molecular species and have homogeneous density without frozen strain, eliminating several sources of uncertainty. This enables the use of established gas models and known properties to accurately predict the acoustic velocity and BFS of gas media, allowing absolute thermometry without the need for calibration, unlike silica fibres. Recent studies^27,28^ have demonstrated that SBS can be highly efficient in gases: in spite of lower densities compared to solids, their much higher compressibility enhances SBS efficiency. Additionally, as the gas density increases, the Brillouin gain increases and the acoustic loss decreases, leading to a quadratic dependence on pressure^21^. By compressing the gas to several tens of bars, the Brillouin gain can exceed that of silica fibres, while maintaining narrower spectral linewidths^21^. Furthermore, lower temperatures further enhance gain and reduce acoustic losses^27,29^, enabling absolute temperature measurements with unprecedented cryogenic sensing performance.

This study identifies optimal gases for absolute thermometry, considering the trade-off between SBS gain, temperature sensitivity, and gas phase transition. Although larger molecular mass yield higher Brillouin gain^21^, complex molecules often have unsuitable molecular absorption lines at wavelengths of interest and liquify at modest pressures and high temperatures, limiting their use. Among practical options, carbon dioxide and nitrogen are promising gases particularly due to their high availability, non-toxicity, and non-flammability. However, lighter gases provide greater BFS sensitivity to temperature^29^, making them more suitable for thermometry, especially at cryogenic temperatures. Additionally, gases must also remain in the gaseous phase at low temperatures, requiring low liquefaction points even under high pressure. These properties are predominantly found in low-mass, simple molecules, such as helium, neon, argon, and nitrogen, which stand out as the most promising candidates for low-temperatures.

Helium, with the lowest mass, could offer excellent sensitivity and remains gaseous down to 5.17 K at 10 bar, being ideal for thermometry at cryogenic temperatures. However, it suffers from very low Brillouin gain and permeates silica easily, requiring a metallic shielding of the fibre, which complicates use. On the other hand, neon liquefies at 37.52 K at 10 bar and remains gaseous down to 44.40 K at 100 bar, showing a liquefication temperature with minimal pressure dependence. This makes it ideal for sensing in the intermediate cryogenic range, particularly near 77 K (i.e., near liquid nitrogen temperatures), relevant for superconductivity and other applications.

At higher cryogenic temperatures, nitrogen and argon are highly suitable: nitrogen liquefies at 103.75 K and argon at 116.60 K, both at 10 bar. Argon, with its higher molecular mass (40 g/mol) compared to nitrogen (28 g/mol) is expected to offer a stronger Brillouin gain, making it ideal for sensing in the range from 120 K to room temperature.

**Fig. 1:**
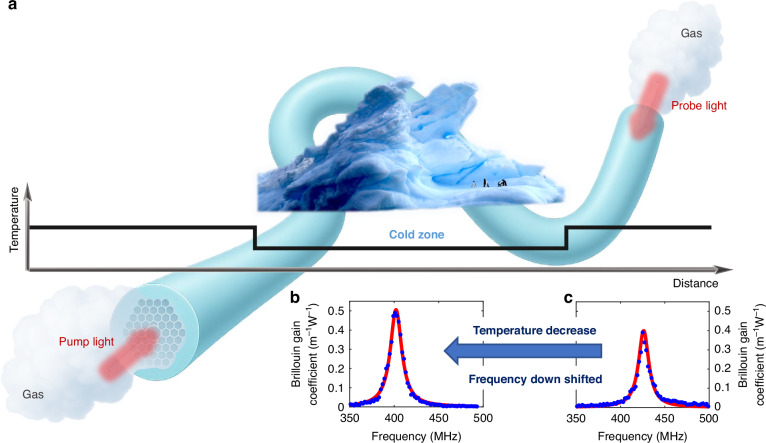
Conceptual diagram of Brillouin-based thermometry using a gas-filled hollow-core fibre (HCF). **a** Pump and probe beams are launched from opposite ends of the HCF, counter-propagating through the gas-filled core. Their interaction generates an acoustic wave via stimulated Brillouin scattering. The HCF is pressurised with gas through both ends to ensure uniform filling. When a segment of the fibre is exposed to a colder environment, the corresponding Brillouin spectrum shifts to lower frequencies compared to that of a segment at ambient temperature. This behaviour is illustrated by the measured spectra for 30 bar argon, showing a downshift at **b** 0 °C relative to (**c**) 23.5 °C. Blue dots represent experimental data, and red curves show Lorentzian fits. The horizontal axis indicates the optical frequency difference between the pump and probe beams, and the vertical axis shows the corresponding Brillouin gain coefficient

To experimentally validate the SBS response of these different gases for absolute thermometry, and to confirm their suitability for cryogenic temperature sensing, we use a gas-filled photonic bandgap hollow-core fibre (HCF) as a waveguide, as illustrated in Fig. 1a. This type of optical fibre allows us to obtain a gas cell for Brillouin scattering analysis, while also offering an extended interaction length under high optical intensities, thereby enhancing measurement accuracy. The high refractive index contrast between the gas-filled core and the surrounding silica, combined with the well-defined photonic bandgap structure, secures a strong light confinement within the gas medium. As a result, the SBS interaction occurs almost entirely within the gas with negligible influence from the silica structure. This ensures that the experimental observations reported herein reflect the intrinsic SBS properties of the gases under investigation.

By scanning the pump-probe frequency difference around the gas BFS, the Brillouin gain spectrum can be accurately measured. As an example, Fig. 1b shows the Brillouin spectrum of argon gas at 30 bar and 0 °C, displaying a Lorentzian shape, characteristic of the dissipative nature of the acoustic wave. The peak frequency represents the BFS and reflects the acoustic velocity in the gas, while the spectral linewidth relates to the acoustic loss. On the other hand, the gain coefficient depends on some gas properties like density and viscosity, which also affect the acoustic attenuation.

It is interesting to notice that the SBS spectral features can only be fully predicted when the underlying acoustic process is well described. In solid-core silica fibres, like in conventional single-mode fibres (SMFs), the acoustic behaviour –representing the collective macroscopic response of all electrostriction-driven dipoles in the medium– is uncertain due to the non-uniform molecular distribution, inhomogeneous density, residual thermal strain, and waveguide geometry variations, causing fibre-to-fibre differences in the acoustic velocity. In contrast, gases exhibit uniform, well-defined thermodynamic properties, allowing precise, model-based predictions of the SBS spectrum and enabling absolute temperature measurements without empirical calibrations. This fundamental distinction highlights the advantages of gas-based SBS sensing demonstrated in this study, compared to solid-core fibre sensors that require individual fibre calibration and only provide relative measurements.

### BFS response in gases for absolute thermometry

Brillouin temperature sensors rely on the monotonic temperature dependence of the BFS. A non-monotonic BFS may yield ambiguous measurements, as multiple temperatures may correspond to the same BFS value.

As stated in Eq. (1), the acoustic velocity ${V}_{a}$ in gases determines the BFS and is given by the Newton–Laplace equation^30^: ${V}_{a}=\sqrt{{\kappa }_{s}/\rho }$, where ${\kappa }_{s}=-V{\left(\partial P/\partial V\right)}_{s}$ is the adiabatic bulk modulus, $P$ is the gas pressure, $V$ is the volume, and $\rho$ is the gas density. The effective refractive index ${n}_{{eff}}$, mainly determined by the gas (due to minimal interaction with the silica structure), is approximated by the gas refractive index *n*, following the Dale-Gladstone relation^31^: $n-1={k}_{{DG}}\rho$, where ${k}_{{DG}}$ is the gas-specific Dale-Gladstone constant^32^ (e.g., ${k}_{{DG}}$ = 74·10^−^^3^ m^3^/kg for neon, 238·10^−^^3^ m^3^/kg for nitrogen and 158·10^−^^3^ m^3^/kg for argon). Combining these, the BFS defined in Eq. (1) becomes:

2 $${\nu }_{B}=\frac{2}{{\lambda }_{0}}\left(1{+k}_{{DG}}\rho \right)\sqrt{\frac{{\kappa }_{s}}{\rho }}$$

It is important to note that optical guiding introduces a small density-independent offset in the effective refractive index, lowering it slightly below 1, with a reduction on the order of ~10^−^^3^ in photonic bandgap fibres and ~10^−4^ in antiresonant HCFs^27,33,34^. While this deviation can introduce a systematic bias in the measured BFS, its effect is generally minimal compared to the practical temperature accuracies. For high-precision scenarios, the effective refractive index in vacuum can be used in place of the unity factor in Eq. (2) for improved accuracy.

Using appropriate gas laws, the absolute BFS can be closely estimated, providing a significant advantage over solid-core fibres, where such predictions are not feasible. The ideal gas law, which treats gas molecules as point particles with no volume and negligible intermolecular forces, is the simplest model for estimating the SBS behaviour in gases, particularly under conditions far from liquification. Described by the state equation^35^ $PV={mRT}$, where $m$ is the number of moles, $R=8.3145{\rm{J}}\cdot {{\rm{K}}}^{-1}\cdot {{\rm{mol}}}^{-1}$ is the universal gas constant and *T* is the absolute temperature, this model reliably describes gas thermodynamics and is suitable for predicting the SBS behaviour. From this, the gas density is $\rho =\frac{P{M}_{m}}{{RT}}$, where ${M}_{m}$ is the molar mass, and the refractive index simplifies to ${n}_{{eff}}={1+k}_{{DG}}\rho =1+\frac{{k}_{{DG}}P{M}_{m}}{{RT}}$. Under the ideal gas model, the bulk modulus is ${\kappa }_{s}=\gamma P$ (see Supplementary Information 1.1), where $\gamma ={C}_{P}/{C}_{V}$ is the heat capacity ratio, with ${C}_{P}$ and ${C}_{V}$ being the specific heat at constant pressure and constant volume, respectively. For monatomic gases, ${C}_{P}=5/2R$ and ${C}_{V}=3/2R$, giving $\gamma =5/3$; while for diatomic gases, ${C}_{P}=7/2R$ and ${C}_{V}=5/2R$, giving $\gamma =7/5$. Note that $\gamma$ remains independent of temperature only if the thermal energy is negligibly transferred to internal molecular states, such as vibrations^35^. As monoatomic gases (e.g., noble gases) lack these internal modes, they offer more stable thermodynamic behavior and are therefore ideal candidates for accurate Brillouin scattering thermometry. The BFS-temperature relationship is thus:

3 $${\nu }_{B}^{\left({ideal}\right)}=\frac{2}{{\lambda }_{0}}\left(1+{k}_{{DG}}\frac{P{M}_{m}}{{RT}}\right)\sqrt{\frac{\gamma {RT}}{{M}_{m}}}$$

**Fig. 2:**
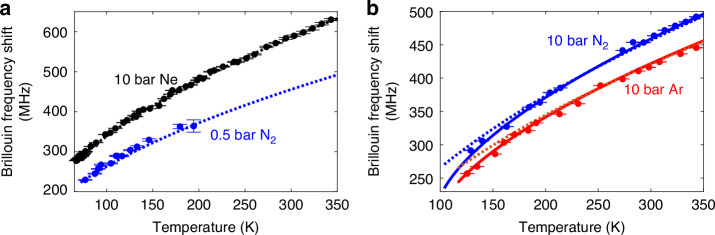
BFS as a function of temperature for various gases at different pressures. **a** BFS response for 10 bar neon (Ne) and 0.5 bar nitrogen (N_2_). Experimental data (dots) are compared with predictions from the ideal gas model (dotted lines). For both gases, the predictions of the ideal gas and van der Waals models coincide, and therefore only the ideal gas model is shown in the figure. Black represents neon and blue represents nitrogen. The measurement for 10 bar Ne is conducted from 70 °C (343.15 K), limited by the thermal resistance of the fibre coating, down to 67 K, the lowest temperature achievable with the available lab cooling setup—but still remaining well above the neon liquefaction temperature of 37.52 K. For 0.5 bar N₂, the temperature range spans from 200 K—limited by the reduced signal amplitude at low gas densities—down to 77 K, which is still higher than its liquefaction temperature of 72 K. The vertical error bars of the experimental data correspond to the standard deviations of the estimation errors (ranging from 0.02 MHz to 3.97 MHz for 10 Bar Ne, and from 0.78 MHz to 15.40 MHz for 0.5 bar N_2_), while the horizontal uncertainty is fixed at 0.26 mK, determined by the accuracy of the temperature measurement. Experimental results show good agreement with the ideal gas model across the entire temperature range, in particular, the prediction for Ne (black dotted line) coincides completely with the experimental data and therefore lies directly beneath the data points. **b** BFS response for 10 bar nitrogen (N₂) and 10 bar argon (Ar). Experimental data (dots) are compared with both ideal gas model predictions (dotted lines) and van der Waals model predictions (solid lines). Blue represents nitrogen and red represents argon. Measurements for 10 bar N₂ range from 343.15 K down to 129 K, remaining above its liquefaction point of 104 K. For 10 bar Ar, data is collected from 350 K to 122 K, above its liquefaction temperature of 117 K. The vertical error bars range from 0.09 MHz to 0.46 MHz for 10 bar N_2_, and from 0.08 MHz to 0.31 MHz for 10 bar Ar, with a constant horizontal uncertainty of 0.26 mK. The van der Waals model accurately predicts the BFS response across the entire temperature range, demonstrating improved agreement over the ideal gas approximation in the denser gas regime at temperatures closer to liquefaction. The lower limit of each theoretical curve corresponds to the liquefaction temperature of the respective gas at the specified pressure. Based on this comparison, the preferred model is chosen in subsequent analyses of the acoustic behaviour of the gas, including the Brillouin linewidth and gain measurements

This expression is largely pressure-independent, as the refractive index additive term is usually small. Interestingly, the BFS described by Eq. (3) is fully predictable when the gas species is known. To verify this, Brillouin measurements have been conducted using nitrogen (N_2_) at 0.5 bar and neon (Ne) at 10 bar. The BFS response versus temperature is shown in Fig. 2a (the way to induce temperature changes in cryogenic conditions is discussed in Supplementary Information 5.1), demonstrating excellent agreement between experimental data (dots) and the BFS estimation using Eq. (3), based on the ideal gas law (dotted lines). However, increasing nitrogen pressure to 10 bar or using argon (Ar) at 10 bar brings their liquification temperature to 104 K and 117 K, respectively, where the ideal gas model becomes no longer valid. As shown in Fig. 2b, the ideal gas model (dotted lines) accurately describes the BFS behaviour above 200 K but diverges at lower temperatures near gas liquification. Indeed, as pressure increases and/or temperature decreases sufficiently, gases approach liquefaction and deviate from ideal behaviour, since the molecular volume becomes a non-negligible fraction of the total volume, making intermolecular forces play an important role^35,36^. As a result, in this regime, the thermodynamic properties must be described using a non-ideal gas model.

The van der Waals equation provides a more accurate description in this non-ideal gas conditions^37^: $(P{M}_{m}^{2}+a{\rho }^{2})({M}_{m}-b\rho )=\rho {RT}{M}_{m}^{2}$, where *a* and *b* are gas-specific van der Waals constants. A closed form for the density $\rho$ is found by solving the resulting cubic equation in $T$ and $P$, as described in Supplementary Information 1. From this model, the bulk modulus at constant entropy *S* is: ${\kappa }_{s}=-V{\left(\frac{\partial P}{\partial V}\right)}_{s}=\gamma \frac{{M}_{m}}{{M}_{m}-b\rho }\left(P+\frac{a{\rho }^{2}}{{{M}_{m}}^{2}}\right)-\frac{2a{\rho }^{2}}{{{M}_{m}}^{2}}$. Substituting this into Eq. (2), the BFS under the van der Waals model becomes^29^:

4 $${\nu }_{B}^{\left({vdW}\right)}=\frac{2}{{\lambda }_{0}}\left(1+{k}_{{DG}}\rho \right)\sqrt{\frac{\gamma }{\rho }\frac{{M}_{m}}{{M}_{m}-b\rho }\left(P+\frac{a{\rho }^{2}}{{{M}_{m}}^{2}}\right)-\frac{2a\rho }{{{M}_{m}}^{2}}}$$

where the density $\rho$ depends on $T$ and $P$, as described in Supplementary Eq. (S3), and $\gamma ={C}_{P}/{C}_{V}$ remains 5/3 for monatomic and 7/5 for diatomic gases. This assumption holds under our experimental conditions, as the HCF is significantly larger than the molecular size and the operating pressure is not extremely high, minimising intermolecular energy contributions and keeping ${C}_{P}$ and ${C}_{V}$ consistent with ideal gas values (see details in Supplementary Information 1.2). Figure 2b validates Eq. (4) using nitrogen and argon at 10 bar. While the ideal gas model fits well above 200 K, the van der Waals model (solid lines) improves accuracy near liquefaction, where deviations arise from non-ideal effects. Note that as the temperature decreases, the BFS slope increases, indicating higher temperature sensitivity at very low cryogenic temperatures. The lowest measurable temperature is determined by the liquefaction point of the gas.

**Fig. 3:**
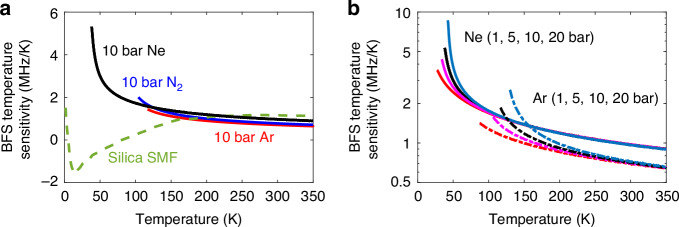
Theoretical BFS thermal sensitivity as a function of temperature for different gases and pressures. **a** Calculated BFS temperature sensitivity for neon (Ne), nitrogen (N₂), and argon (Ar), each at a pressure of 10 bar. For comparison, the BFS thermal sensitivity of a conventional silica SMF is shown as a green dashed line, obtained based on experimental measurements. Unlike gases, silica SMF exhibits a markedly different trend, with significantly reduced sensitivity at cryogenic temperatures. **b** Calculated BFS thermal sensitivity of Ne and Ar at pressures of 1, 5, 10, and 20 bar, plotted across their respective temperature ranges. The lower end of each curve corresponds to the liquefaction temperature of the gas at the given pressure. The plots highlight how the gas type, pressure, and absolute temperature collectively influence the BFS thermal sensitivity

Figure 3a compares the BFS temperature sensitivity in MHz/K (calculated in Supplementary Information 3) for neon, nitrogen, and argon at 10 bar, alongside that of a silica SMF^8–10^ (green dashed line, calculated based on Supplementary Information 4). Results point out that lighter gases (e.g., neon), while offering slightly higher sensitivity at elevated temperatures, are particularly suited for cryogenic temperature sensing since they remain gaseous where heavier gases tend to liquify. In contrast, heavier gases (e.g., argon) can surpass the response of lighter gases below ∼150 K due to their enhanced sensitivity provided near liquefaction. It is worth noticing that gas-based SBS sensing offers enhanced sensitivity below ∼120 K compared to SMFs, which show no response around 80 K, enabling temperature measurements in ranges inaccessible to conventional SMF-based SBS sensors.

Figure 3b shows the impact of pressure on the temperature sensitivity of neon and argon. Results point out that increasing the gas pressure enhances the temperature sensitivity due to the higher gas density, though it also raises the minimum measurable temperature, as the liquification temperature increases. The highest sensitivity occurs at the liquefaction point, a trend confirmed across all analysed gases. These theoretical predictions are validated experimentally in Fig. S3 (see details in Supplementary Information 4), where calculated sensitivities for different gases match well those obtained from numerical differentiation of BFS measurements in Fig. 2. Results confirm that gas-based SBS sensing at cryogenic temperatures can reach sensitivities up to 9 times (e.g., for neon at 20 bar) larger than SMFs at room temperature (typically ∼1 MHz/K), highlighting its superior precision. This enhanced sensitivity is even more significant when compared to the poor, near-zero sensitivity of SMFs around 80 K, as shown in Fig. 3a.

### Temperature uncertainty in gases: impact of Brillouin linewidth and gain

Note that the precision of the BFS determination, defining the temperature resolution, depends on the measurement signal-to-noise ratio (SNR) and the Brillouin spectral linewidth^38^. When using a quadratic fitting of the spectral points over the full width at half maximum (FWHM) of the Brillouin spectrum for BFS extraction, the frequency uncertainty ${\sigma }_{{\nu }}$ is given by^38^:

5 $${\sigma }_{{\rm{\nu }}}=\frac{1}{{SNR}}\sqrt{\frac{3}{4}\delta \cdot \Delta {\nu }_{B}}$$

where ${SNR}$ is the signal-to-noise ratio at the peak frequency, $\delta$ is the spectral sampling step, and $\Delta {\nu }_{B}$ is the Brillouin spectral FWHM. The temperature resolution ${\sigma }_{T}$ is therefore defined as

6 $${\sigma }_{T}=\frac{{\sigma }_{{\rm{\nu }}}}{d{\nu }_{{\rm{B}}}/{dT}}$$

where $\frac{d{\nu }_{{\rm{B}}}}{{dT}}$ represents the temperature sensitivity, as described in Supplementary Eqs. (S19) and (S20). Alternative curve-fitting approaches, such as Gaussian, Lorentzian or Voigt profiles, are expected to yield similar dependences on these parameters, differing only in the constant factor^38^. Note that, while the impact of SNR on the BFS precision is intuitive, narrower Brillouin linewidths also lead to more accurate BFS determination. Therefore, a comprehensive analysis of both the Brillouin linewidth and Brillouin gain coefficient (which determines the spectral peak amplitude and SNR) is key to assessing the temperature resolution of the proposed approach.

The Brillouin linewidth is directly proportional to the acoustic attenuation coefficient, primarily determined by viscous forces and the thermal conductivity of the interacting medium, and can be described as^39^:

7 $${\varDelta \nu }_{B}\left(T,P\right)=\frac{{q}^{2}{\eta }_{s}}{2\pi \rho }\left(\frac{4}{3}+{c}_{\eta }+\frac{\gamma -1}{{P}_{r}}\right)$$

where $q\approx 2n\omega /c$ is the acoustic wavenumber, ${{\eta }}_{s}$ is the shear viscosity, ${c}_{\eta }$ is a gas-dependent constant^40^ (0 for inert gases, and ∼0.67 for diatomic gases), and ${P}_{r}$ is the Prandtl number^41^. Supplementary Information 2.1 details the full model, including both ideal and non-ideal gas corrections.

**Fig. 4:**
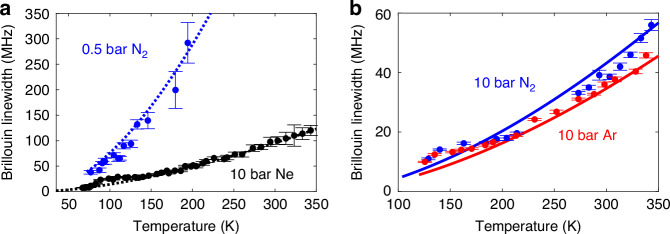
Brillouin linewidth as a function of temperature for various gases at different pressures. **a** Brillouin spectral linewidth for 10 bar neon (Ne) and 0.5 bar nitrogen (N₂). Experimental data (dots) are compared with predictions from the ideal gas model (dotted lines), presented in Supplementary Eq. (S13). Black represents neon and blue represents nitrogen. The measurements for 10 bar Ne are conducted from 343.15 K down to 67 K, remaining well above the neon liquefaction temperature of 37.52 K. For 0.5 bar N₂, the temperature range spans from 200 K down to 77 K, which is still higher than its liquefaction temperature of 72 K. The vertical error bars of the experimental data correspond to the standard deviations of the estimation errors (ranging from 0.08 MHz to 21.00 MHz for 10 Bar Ne, and from 2.83 MHz to 39.70 MHz for 0.5 bar N_2_), while the horizontal uncertainty is fixed at 0.26 mK, determined by the accuracy of the temperature measurement. Experimental Brillouin linewidths show good agreement with the theoretical predictions based on the ideal gas model across the entire temperature range. **b** Brillouin spectral linewidth for 10 bar nitrogen (N₂) and 10 bar argon (Ar). Experimental data (dots) are compared with van der Waals model predictions (solid lines), based on Supplementary Eq. (S14). Blue represents nitrogen and red represents argon. Measurements for 10 bar N₂ range from 343.15 K down to 129 K, remaining above its liquefaction point of 104 K. For 10 bar Ar, data is collected from 350 K to 122 K, above its liquefaction temperature of 117 K. The vertical error bars range from 0.24 MHz to 1.84 MHz for 10 bar N_2_, and from 0.19 MHz to 0.88 MHz for 10 bar Ar, with a constant horizontal uncertainty of 0.26 mK. The figure demonstrates that the van der Waals model in Supplementary Eq. (S14) accurately predicts the Brillouin linewidth response across the entire temperature range. The lower limit of each theoretical curve corresponds to the liquefaction temperature of the respective gas at the specified pressure

Figure 4a presents the Brillouin linewidth measured from 67 K to 350 K for 0.5 bar nitrogen and 10 bar neon. while Fig. 4b shows the linewidth for nitrogen and argon at 10 bar, just above their liquification. Based on the model comparison in Fig. 2, the theoretical linewidth in Fig. 4a is calculated using the ideal gas model, whereas in Fig. 4b it is derived from the van der Waals model. Results confirm that, unlike in solid-core silica fibres, where linewidth broadens as temperature decreases down to about 100 K^9^, the SBS linewidth in gases decreases monotonically with temperature. This is due to the increasing gas density at lower temperatures, which reduces attenuation, resulting in narrower linewidths and, thus, better BFS precision at cryogenic temperatures. In addition, Fig. 4 also validates the accuracy of the theoretical models presented in Supplementary Information 2.1 (dotted and solid lines), which closely match the experimental data (dots), even near liquification. Small systematic deviations observed between 100 K and 150 K are attributed to the spectral broadening caused by thermal gradients along the fibre inside the dewar used in the experiment (see Methods).

Another key parameter influencing the quality of SBS measurements, and thus the precision of SBS-based thermometry, is the Brillouin gain coefficient. A higher gain coefficient leads to an improved SNR at the peak frequency, enhancing the precision of BFS estimations^38^. The Brillouin gain coefficient^21^, which is the material linear gain ${g}_{B}$ divided by the effective area ${A}_{{eff}}^{{ao}}$, can be simplified, assuming that the electrostrictive constant of the gas is ${\gamma }_{e}=({n}^{2}-1)({n}^{2}+2)/3\approx 2{k}_{{DG}}\rho$, so that:

8 $${\gamma }_{B}=\frac{{g}_{B}}{{A}_{{eff}}^{{ao}}}=\frac{{\gamma }_{e}^{2}{\omega }^{2}}{\rho n{V}_{a}{c}^{3}2\pi \varDelta {\nu }_{B}{A}_{{eff}}^{{ao}}}\approx \frac{16\pi {k}_{{DG}}^{2}\rho }{{\lambda }_{0}^{3}c{\nu }_{B}\varDelta {\nu }_{B}{A}_{{eff}}^{{ao}}}$$

where *c* is the speed of light in vacuum, ${A}_{{eff}}^{{ao}}$ is the acousto-optic overlap effective area (equal to 80 μm^2^ for our HCF), and $\omega$ is the optical angular frequency.

**Fig. 5:**
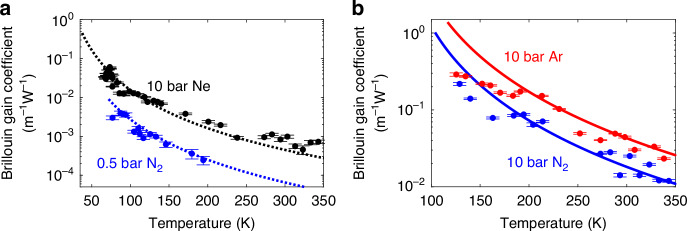
Brillouin gain coefficient as a function of temperature for various gases at different pressures. **a** Brillouin gain for 10 bar neon (Ne) and 0.5 bar nitrogen (N₂). Experimental data (dots) are compared with predictions from the ideal gas model (dotted lines), presented in Supplementary Eq. (S16). Black represents neon and blue represents nitrogen. The measurements for 10 bar Ne are conducted from 343.15 K down to 67 K, remaining well above the neon liquefaction temperature of 37.52 K. For 0.5 bar N₂, the temperature range spans from 200 K down to 77 K, which is still higher than its liquefaction temperature of 72 K. The vertical error bars of the experimental data correspond to the standard deviations of the estimation errors (ranging from $7.40\times {10}^{-5}{{\rm{m}}}^{-1}{{\rm{W}}}^{-1}$ to $6.10\times {10}^{-3}{{\rm{m}}}^{-1}{{\rm{W}}}^{-1}$ for 10 Bar Ne, and from$6.08\times {10}^{-5}{{\rm{m}}}^{-1}{{\rm{W}}}^{-1}$ to $4.82\times {10}^{-4}{{\rm{m}}}^{-1}{{\rm{W}}}^{-1}$ for 0.5 bar N_2_), while the horizontal uncertainty is fixed at 0.26 mK, determined by the accuracy of the temperature measurement. Experimental SBS gain coefficients show good agreement with the theoretical predictions based on the ideal gas model across the entire temperature range. **b** Brillouin gain for 10 bar nitrogen (N₂) and 10 bar argon (Ar). Experimental data (dots) are compared with van der Waals model predictions (solid lines), based on Supplementary Eq. (S17). Blue represents nitrogen and red represents argon. Measurements for 10 bar N₂ range from 343.15 K down to 129 K, remaining above its liquefaction point of 104 K. For 10 bar Ar, data is collected from 350 K to 122 K, above its liquefaction temperature of 117 K. The vertical error bars range from 4.27$\times {10}^{-4}{{\rm{m}}}^{-1}{{\rm{W}}}^{-1}$ to $9.99\times {10}^{-3}{{\rm{m}}}^{-1}{{\rm{W}}}^{-1}$ for 10 Bar N_2_, and from $5.04\times {10}^{-4}{{\rm{m}}}^{-1}{{\rm{W}}}^{-1}$ to $1.46\times {10}^{-2}{{\rm{m}}}^{-1}{{\rm{W}}}^{-1}$ for 10 bar Ar, with a constant horizontal uncertainty of 0.26 mK. The figure demonstrates that the van der Waals model in Supplementary Eq. (S17) accurately predicts the Brillouin gain coefficient across the entire temperature range. The lower limit of each theoretical curve corresponds to the liquefaction temperature of the respective gas at the specified pressure

Figure 5 shows the temperature dependence of the Brillouin gain coefficient, measured for 0.5 bar nitrogen, 10 bar neon, 10 bar nitrogen, and 10 bar argon. Based on the model comparison in Fig. 2, experimental results are compared to theoretical predictions under ideal gas conditions (Fig. 5a) and near liquification (Fig. 5b) between 67 K and 350 K. Details on how the SBS gain coefficients are extracted from the measurements, including parameter conversions, are provided in the Supplementary Information 2.3. At ambient pressure and temperature, the Brillouin linewidth in gas is broader than in standard single-mode fibres (SMFs), resulting in a lower Brillouin gain factor. However, increasing the gas pressure or lowering the temperature raises the gas density, significantly amplifying the Brillouin response, as indicated by the results. Under sufficiently high pressure or low temperature, the linewidth in a gas-filled hollow-core fibre can become narrower than in SMF, with the Brillouin gain factor even surpassing that of SMFs^21^. This amplitude enhancement boosts SNR at cryogenic temperatures, further supporting the use of SBS in gaseous media for precise cryogenic thermometry. Experimental data (dots) show strong agreement with theoretical predictions (dotted and solid lines), validating the models based on the ideal and non-ideal gas behaviours presented in the Supplementary Information 2.2. Note that temperature inhomogeneities around 150 K, as already described for the spectral broadening in Fig. 4, also lower the measured amplitude, resulting in a slight underestimation of the experimental Brillouin gain coefficient in Fig. 5b around 150 K.

**Fig. 6:**
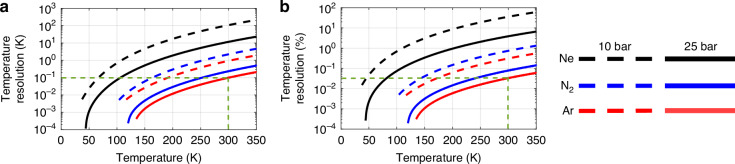
Estimated temperature resolution in gas-based Brillouin thermometry. **a** Absolute temperature resolution in Kelvin, and (**b**) relative temperature resolution (as a percentage of the operating temperature), plotted as functions of temperature for the three studied gases—neon (Ne), nitrogen (N₂), and argon (Ar)—at pressures of 10 bar and 25 bar. The green dashed line indicates a reference resolution of 0.1 K, corresponding to a typical performance of Brillouin-based temperature sensors in conventional silica single-mode fibres at room temperature (300 K)

Note that the presented analysis highlights the strong temperature dependence of Brillouin scattering in gases, including the spectral narrowing and increased gain coefficient at cryogenic temperatures, enhancing the SBS efficiency and reducing the BFS uncertainty compared to room-temperature measurements. Along with the higher BFS sensitivity at low temperatures shown in Fig. 3, these effects make SBS-based temperature sensing in gases a superior alternative to conventional Brillouin sensors in silica fibres, which exhibit reduced and non-monotonic temperature sensitivity in cryogenic conditions^8–10^. By combining Eqs. (5) and (6) with the proposed models for the temperature dependence of the Brillouin linewidth, gain coefficient, and BFS sensitivity, the absolute temperature resolution can be estimated, assuming a known detection noise. For instance, considering a reference resolution of 0.1 K (a common level considered in literature as a benchmark for high-performance Brillouin systems) at room temperature in silica to define a baseline measurement noise, Fig. 6 shows the theoretical estimated temperature resolution, as a function of the operating temperature for the three analysed gases at 10 bar (dashed lines) and 25 bar (solid lines). Results confirm that SBS in gases offers exceptional temperature resolutions and a significant advantage at cryogenic temperatures where SMFs perform poorly. Furthermore, SBS in gases remains effective at room temperature: for instance, argon at 25 bar achieves a comparable temperature resolution to SMF-based systems (green dashed line). The figure also shows that increasing the gas pressure, which in turn raises its density, strengthens the Brillouin interaction and reduces acoustic attenuation, leading to a higher gain coefficient and a narrower linewidth, both improving the temperature resolution. The next section further analyses the influence of the gas pressure on the SBS spectral response and temperature resolution.

### Impact of the gas pressure on the Brillouin response and temperature uncertainty

**Fig. 7:**
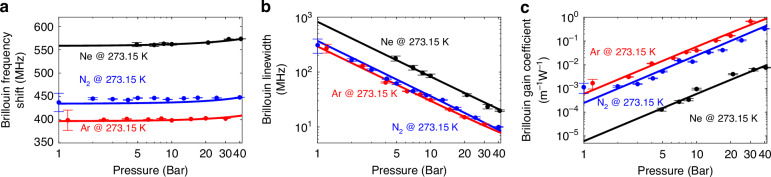
Impact of the gas pressure on the Brillouin spectral response at 0 °C (273.15 K). **a** Brillouin frequency shift, **b** Brillouin linewidth, and **c** Brillouin gain coefficient are plotted as functions of the gas pressure for nitrogen (N₂, blue), argon (Ar, red), and neon (Ne, black). Experimental data points (dots) are shown alongside theoretical predictions (solid lines) based on the van der Waals gas model. The vertical error bars of the experimental data correspond to the standard deviations of the estimation errors, with ranges as follow: (**a**) BFS - 0.05 MHz to 19.80 MHz (N_2_), 0.10 MHz to 21.79 MHz (Ar), and 0.20 MHz to 4.34 MHz (Ne); **b** Brillouin linewidth - 0.20 MHz to 89.60 MHz (N_2_), 0.28 MHz to 49.82 MHz for (Ar), and 0.636 MHz to 18.70 MHz for (Ne); **c**. Brillouin gain coefficient - $6.92\times {10}^{-5}{{\rm{m}}}^{-1}{{\rm{W}}}^{-1}$ to $7.70\times {10}^{-3}{{\rm{m}}}^{-1}{{\rm{W}}}^{-1}$ (N_2_), 1.32$\times {10}^{-4}{{\rm{m}}}^{-1}{{\rm{W}}}^{-1}$ to $2.01\times {10}^{-2}{{\rm{m}}}^{-1}{{\rm{W}}}^{-1}$ (Ar), and $1.87\times {10}^{-5}{{\rm{m}}}^{-1}{{\rm{W}}}^{-1}$ to $7.43\times {10}^{-4}{{\rm{m}}}^{-1}{{\rm{W}}}^{-1}$ (Ne). The horizontal uncertainty is fixed at 0.01 bar, determined by the gas pressure meter accuracy. The figure illustrates how increasing gas pressure affects each Brillouin parameter, highlighting differences in behaviour among the three gases

Under ambient conditions, the Brillouin gain coefficient in gases is relatively low^21^, which can result in high temperature uncertainties. As shown in Fig. 6, increasing the gas density —taking advantage of the natural compressibility of gases— enhances the Brillouin interaction, thereby reducing BFS uncertainty and improving temperature resolution, both at room temperature and in cryogenic regimes. To analyse how SBS spectral characteristics respond to pressure changes, Fig. 7 presents the measured BFS, Brillouin linewidth, and gain coefficient of neon, nitrogen, and argon at 0 °C (273.15 K) as a function of the gas pressure. Note that in all experiments the gas pressure was kept below 50 bar, consistent with the safe operating range of our laboratory setup. This value is far from the actual pressure limit of hollow-core fibres. In fact, the literature reports that hollow-core photonic crystal fibres can withstand pressures up to 1000 bar^42^, confirming that the operating pressures required for reliable sensing remain well within safe margins.

As shown in Fig. 7a, the BFS exhibits only a slight dependence on the gas pressure, mainly affected by the small pressure-induced variations in the gas refractive index, as described by Eq. (2). In contrast, pressure has a much stronger impact on the Brillouin linewidth (Fig. 7b) and gain coefficient (Fig. 7c). As gas pressure, and therefore gas density, increases, the acoustic attenuation reduces and the Brillouin interaction strengthens, resulting in narrower linewidths and higher gain coefficients. These responses confirm that by adjusting the gas pressure, the SBS sensing performance can be optimised for specific temperature ranges, while impacting negligibly the temperature determination.

Additionally, the gas molar mass also significantly influences the SBS spectral response. Lighter gases yield higher acoustic resonance frequencies and thus higher BFS values. For example, neon (20 g/mol) shows the highest BFS, followed by nitrogen (28 g/mol) and then argon (40 g/mol). Although higher molar mass tends to reduce acoustic attenuation and enhance SBS gain, other thermodynamic properties, such as the gas viscosity, also play a key role. Argon, despite its greater molar mass, is 1.25 times more viscous than nitrogen, increasing acoustic losses and offsetting its expected advantage. As a result, argon and nitrogen exhibit nearly identical linewidths and gain coefficients. This highlights that SBS spectral characteristics are governed by a complex interplay of parameters, and not only by a single factor.

**Fig. 8:**
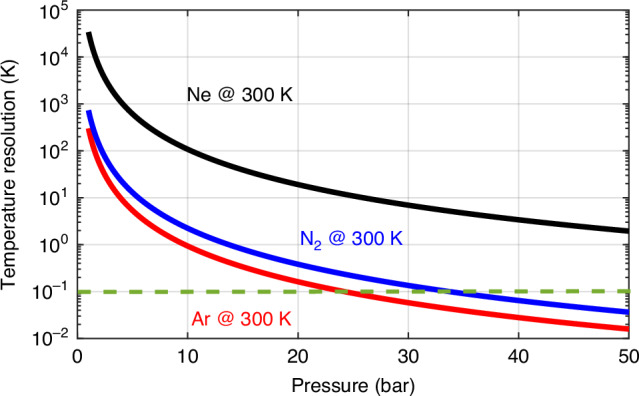
Estimated SBS-based temperature resolution versus pressure at 300 K for different gases. The theoretical temperature resolution is evaluated for neon (Ne), nitrogen (N₂), and argon (Ar) across a range of pressures at an operating temperature of 300 K. The green dashed line indicates the reference resolution of 0.1 K typically achieved with Brillouin-based sensors in conventional silica SMFs. The results demonstrate that increasing the gas pressure—hence the gas density—enhances the temperature resolution of the proposed SBS-based thermometry in gases

Incorporating the pressure-dependent behaviour of the SBS response into Eqs. (5) and (6) allows us to predict the temperature resolution as a function of gas pressure. Figure 8 indicates that increasing the pressure significantly improves the temperature resolution, making gas-based SBS thermometry viable even at ambient conditions. This analysis, carried out at 300 K, assumes a system noise level leading to a 0.1 K resolution in standard SMF-based sensors (green dashed line).

### Gas-based distributed Brillouin temperature sensing

**Fig. 9:**
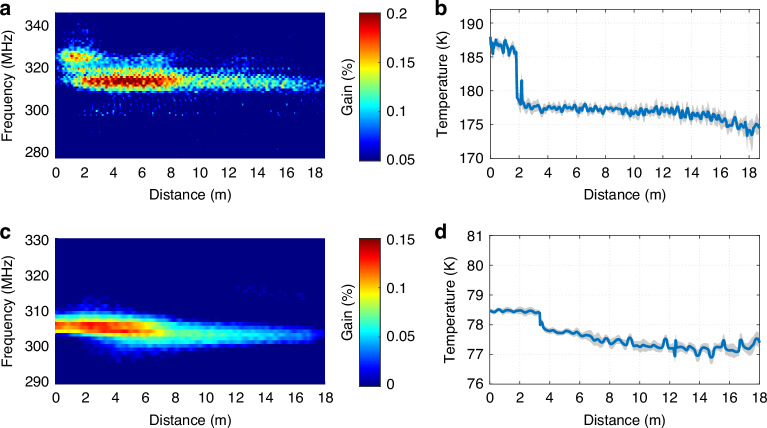
Experimental demonstration of distributed SBS-based thermometry in gases. **a** Top-view of the Brillouin gain spectrum along the HCF with (**b**) the corresponding distributed temperature profile, obtained with a spatial resolution of 45 cm using 18.5 bar of argon at temperatures below 200 K, exhibiting an average accuracy of 0.29 K (maximum value of 0.79 K near the fibre end). The grey shaded region denotes the ±3σ uncertainty around the estimated central value (blue curve), calculated using Eqs. (5)-(6) and corresponding to the 99.7% confidence interval. A localised heated section of approximately 1.5 m near the beginning of the HCF clearly appears as a temperature hotspot in the measured profile. **c** Top-view of the Brillouin gain spectrum along the HCF with **d** the corresponding distributed temperature profile, obtained with a spatial resolution of 75 cm using 27 bar of neon around 77 K, exhibiting an average accuracy of 0.050 K (maximum value of 0.093 K near the fibre end). The grey shaded region denotes the ±3σ uncertainty around the estimated central value (blue curve). A localised hotspot of about 3 m near the beginning of the HCF is clearly observed

At room temperature, the gas-based backward Brillouin scattering temperature sensing scheme has already been demonstrated by using high pressure gases^21^. To demonstrate the capability of distributed absolute temperature sensing in the cryogenic range, a time-domain Brillouin echoes interrogation approach^43^ has been implemented to measure the local Brillouin response along an 18.5 m-long HCF filled successively with argon at 18.5 bar and neon at 27 bar, at temperatures below 200 K. Experimental details are provided in the Methods section. First, the measurements are performed using argon: for this, 17 m of the HCF are placed about 10 cm above the liquid nitrogen surface (at ∼177 K), while a 1.5 m section near the fibre input (where the pump is launched) is placed about 4 cm higher (at ∼187 K). A 3 ns $\pi$-phase pump pulse is used to obtain the time-domain response of the Brillouin gain along the HCF with a spatial resolution of 45 cm. A sampling rate of 5 GS/s is used, corresponding to a spatial sampling step of 6 cm, allowing for an accurate spatial representation of the measurand. Figure 9a shows the distributed Brillouin gain spectrum, where the gain is expressed as a percentage of the probe light amplification. Figure 9b presents the extracted temperature profile along the sensing fibre, which is obtained through quadratic fitting of the local gain spectra, with corresponding temperature uncertainty derived from Eqs. (5) and (6) ^38^. Then, for cryogenic temperature sensing, the HCF is filled with neon: 15 m of it are fully immersed in liquid nitrogen (at ∼77 K), while a 3 m section near the fibre input end is suspended < 1 cm above the liquid nitrogen surface, remaining about 1–2 K warmer. A 5 ns $\pi$-phase pump pulse is used in this case to provide a spatial resolution of 75 cm, while keeping the spatial sampling of 6 cm. Figure 9c, d show the distributed Brillouin gain spectrum and the corresponding temperature profile along the fibre.

In the distributed measurements, we assume that the relationship between BFS and temperature as validated in Section 2.2, are fully transferable to the distributed measurements. A distortion of the Brillouin gain spectrum is not anticipated, since non-local effects such as pump depletion^44^ and frequency shifts arising from self-phase modulation^45^ are calculated to be entirely negligible under the present conditions. These measurements results confirm the distributed temperature sensing capabilities of Brillouin scattering in gases, especially under cryogenic environments. It is important to mention that the temperature profile in Fig. 9 is directly derived from the measured BFS using the van der Waals gas model, without empirical BFS-to-temperature calibrations typically required in silica-based Brillouin sensors. This demonstrates a key advantage of the proposed gas-based Brillouin sensing approach, providing accurate, calibration-free, and highly sensitive absolute temperature measurements across a wide range, including deep cryogenic regimes where silica-based systems fail.

## Discussion

In this study, we characterise the Brillouin scattering response in gases to assess its viability as a thermometric method, focusing on gas species and pressure.

Our experimental setup employs flexible gas feedthroughs to fill the hollow core of the fibre, which is effective for controlled laboratory experiments, but impractical for real-world use. To address this, Supplementary Information 5.2 describes an alternative configuration with pre-filled HCFs spliced with SMFs, ensuring reliable light guidance and a secure, gas-sealed environment, suitable for practical applications. To further reduce the minimum measurable temperature of gas-based Brillouin thermometry, the sensing medium must remain in the gaseous phase. This strongly favours gases with low liquefaction temperatures, particularly for cryogenic sensing below 77 K (the boiling point of liquid nitrogen^46^). In such scenarios, low-density gases are especially well-suited. As illustrated in Figs. 2, 3, and 6, neon is ideal for cryogenic sensing above 27 K (its liquefaction temperature at 1 bar). At 40 bar and 77 K, the Brillouin gain coefficient of neon (0.552 m^−1^W^−1^) is approximately twice that of SMFs at room temperature, enabling a high SNR and precise temperature measurements.

In general, the minimum measurable temperature can be adjusted either by changing the gas species or by varying the pressure; however, this involves a trade-off. For a given gas, lowering the pressure decreases the liquefaction temperature, extending the measurable range toward lower values. However, this also reduces the Brillouin gain, which lowers the SNR and increases the temperature uncertainty. This limitation can be mitigated by switching to a lighter gas: although lighter gases naturally provide lower gain, the effect can be compensated by operating at higher pressures, still enabling a reduced liquefaction temperature and thus an extended measurement range.

**Fig. 10:**
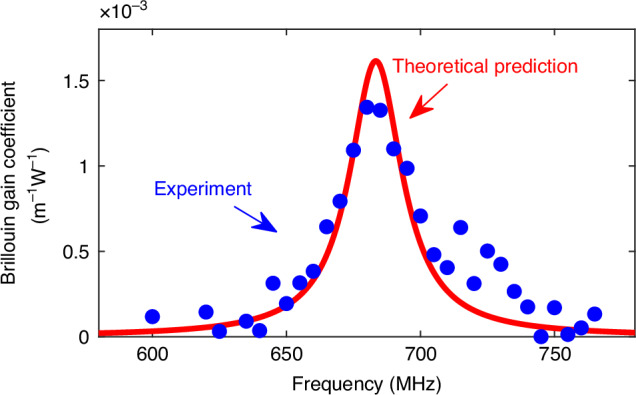
Brillouin gain spectrum of helium at 77 K and 14 bar. Blue dots represent the experimentally measured spectrum, while the red curve shows the corresponding theoretical prediction based on the ideal gas model. The measured BFS of 685.5 MHz closely matches the theoretically predicted value of 683.37 MHz, confirming the accuracy of the SBS spectral model under cryogenic conditions. These results also demonstrate the feasibility of using helium as a Brillouin interacting medium for absolute thermometry at deep cryogenic temperatures

For ultra-low temperatures, helium is preferred due to its exceptionally low liquefaction point of 4 K at 1 bar^46^, estimated as the lowest achievable temperature point. Helium exhibits a Brillouin gain roughly 50 times lower than that of neon, as estimated from Eq. (8) and verified experimentally hereafter, and easily permeates through silica^47,48^, with full diffusion occurring within tens of minutes as observed in our fibre. To address this issue, a preliminary experiment has been conducted at 77 K using helium at 14 bar, with the HCF encased in a metallic capillary to reduce gas leakage. This approach extended the system stability to approximately 2 h before any noticeable signal degradation occurred. The measured Brillouin gain spectrum in Fig. 10 confirms the feasibility of helium-based Brillouin measurements for cryogenic temperature sensing, verifying a clear gain peak at the tested temperature. Further experiments targeting 4 K are planned, although our progress is limited by the available cooling infrastructure in the laboratory.

As previously described, Brillouin scattering in gases is fundamentally governed by the thermodynamic behaviour of the medium, enabling the development of a novel, absolute temperature scale based on gas-phase SBS. Noble gases are particularly well-suited for this purpose, as their specific heat ratio $\gamma$ remains essentially constant over a wide temperature range^35^. This property simplifies the relationship between the BFS and temperature. In addition, under ideal gas conditions, noble gases exhibit refractive indices very close to one^32^, with deviations below 0.01% per bar (e.g. for neon, calculated based on Dale-Gladstone relation^31^), negligible for most applications.

Under these assumptions, the BFS is given by: ${\nu }_{B}=\frac{2}{{\lambda }_{0}}\sqrt{\frac{\gamma {RT}}{{M}_{m}}}$. This equation highlights that ${\nu }_{B}$ is independent of pressure and scales with the square root of temperature, making the method inherently absolute and highly reliable for thermometric applications. For higher precision, the refractive index can be calibrated using the known gas pressure.

When confined within an HCF, gas-based Brillouin sensing serves as an effective contact thermometer—suitable for a wide range of practical scenarios. However, for applications requiring contactless temperature measurements—such as sensitive cryogenic environments or materials with delicate thermal profiles—gas-based Brillouin thermometry offers a promising contactless, non-invasive solution. Since the SBS spectral features measured in our work with a gas-filled fibre are dominated by the gas response, with negligible contribution from the surrounding silica, thus similar Brillouin responses are expected to be observed in free-space configurations.

Unlike conventional contactless thermometry methods based on blackbody radiation, which suffer from limited accuracy and significantly reduced sensitivity at cryogenic temperatures, Brillouin thermometry offers high accuracy and minimal thermal disturbance. Employing free-space Brillouin spectroscopy^18,19^, this approach can be extended into a versatile, absolute, and contactless thermometer, introducing minimal energy into the probed medium, thereby minimising thermal disturbance. A key challenge lies in the requirement for high-pressure gas conditions to achieve significant Brillouin gain at room temperature. This limitation, however, could be alleviated at cryogenic temperatures, where higher Brillouin gain can naturally be obtained.

Actually, the energy deposited through the phonon generation and dissipation during the SBS process is exceptionally low (which we estimate to be less than 1 nW, based on the scattering process) since each scattered photon corresponds to the creation of a single acoustic phonon. This ultra-low energy exchange represents a significant advantage over conventional thermometry, especially in applications where even minor heating must be avoided.

Altogether, these advantages make gas-based Brillouin thermometry a foundational tool for redefining temperature measurements with unprecedented precision, versatility, and minimal invasiveness, being particularly suited for demanding cryogenic and high-precision scientific environments.

## Methods

### Experimental setup for Brillouin spectral measurements in gases

**Fig. 11:**
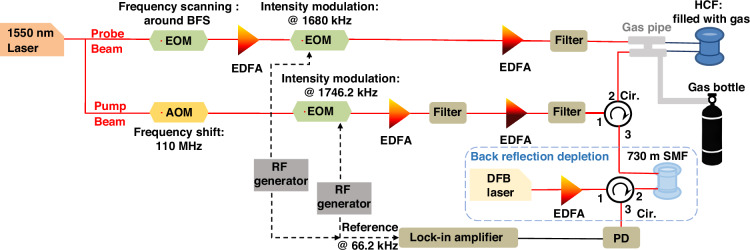
Experimental setup for measuring the SBS response in a gas-filled HCF. The system enables the measurement of the integrated (average) Brillouin response along the full length of an HCF filled with gases at different pressures and exposed at varying temperatures. A double-modulation scheme is applied to the pump and probe sidebands, while the detection is performed using a lock-in amplifier. To suppress strong back-reflections of the Brillouin pump, originating from the SMF-HCF input interface, a back-reflection depletion stage is incorporated. This stage uses Brillouin loss to generate a narrow optical notch filter ( ~ 10 MHz) that effectively attenuates the reflected pump signal

Figure 11 illustrates the experimental setup used to measure the Brillouin spectrum in the different analysed gases. Light from a 1550 nm external cavity laser with a linewidth of 23 kHz is split by an optical coupler into two branches to generate both the pump and probe lights. In the upper branch of the figure, a dual-sideband probe with suppressed carrier is generated using a Mach-Zehnder electro-optic modulator (EOM) modulated by a microwave generator at an adjustable frequency ${f}_{{scan}}$. On the other (lower) branch, an acousto-optic modulator (AOM) is used to upshift the laser optical frequency by ${f}_{{AOM}}$ = 110 MHz. This breaks the spectral symmetry between the pump and the two probe sidebands, ensuring that only the lower-frequency probe sideband participates in the Brillouin interaction. As a result, only the Brillouin gain spectrum can be measured even when both probe sidebands are detected by the photo-detector (PD) simultaneously without the use of an optical filter before photodetection. Note that this configuration is required because the frequency difference between the sidebands is around 1 GHz or lower, given that that BFS values of the analysed gases are only a few hundred MHz. To enhance the measurement sensitivity, a dual intensity modulation scheme^21^ is applied, in which independent EOMs modulate the pump and probe at 1680 kHz and 1746.2 kHz, respectively. Insertion losses are compensated by erbium-doped fibre amplifiers (EDFAs) in both branches, enabling input powers of 30 dBm for the pump and 4 dBm for the probe at the gas-filled HCF. The setup employs a 20 m photonic bandgap HCF as a single-mode waveguide and gas confinement medium, providing an acousto-optic overlap effective area of 80 µm^2^ and a total optical propagation loss of 6.5 dB (slightly increased due to a previous long-term exposure to water vapour in the atmosphere).

Note that the dual intensity modulation method^21^ is here used to enhance the measurement sensitivity, enabling SBS detection even under gas temperature and pressure conditions where the Brillouin gain is several orders of magnitude lower than in conventional SMFs. This method introduces a beating Brillouin component in the SBS interaction at the sum and difference frequencies of the intensity modulations (i.e., 66.2 kHz and 3426.2 kHz, in this case). A lock-in amplifier synchronised with the intensity modulation clock signals is used to measure the SBS response at the 66.2 kHz component, which exhibits lower noise compared to the 3426.2 kHz component. To ensure consistent and comparable results across all experiments, this double lock-in detection scheme is applied throughout the study. This approach is particularly important since this work aims to characterize the Brillouin response over a broad range of conditions, including non-heavy gases at low pressures where the SBS gain is intrinsically weak. The measurement time for each data point is determined as a fixed multiple (according to standard methodology) of the lock-in amplifier time constant. This constant was set to a minimum of 0.3 s, and increased for cases with lower SBS gain (e.g., at lower pressures or higher temperatures), to maintain a relatively uniform measurement error across the full temperature range

The Brillouin spectral response is then measured with very high sensitivity by scanning the detuning frequency ${f}_{{scan}}-{f}_{{AOM}}$ around the BFS, and is extracted by performing a curve fitting on the scanning spectrum, which is further discussed in Supplementary Information 6.

To secure both optical transmission and gas flow, the HCF is butt-coupled to angle-cleaved SMF patch cords at both ends, leaving ~10 µm gaps to allow gas to flow into the optical fibre. This coupling introduces about 6 dB of loss at each interface. The coupling region is enclosed in a gas-tight system connected to gas bottles. Once the fibre is completely filled with gas, the system remains stable for all tested gases except helium. In the case of helium, rapid permeation through the silica occurs within about 10 min, making the use of a metallic capillary necessary to maintain stability. One of the key challenges in the setup is the reflection of the Brillouin pump launched into the HCF at the SMF cleaving surface, caused by refractive index mismatch. Although angle-cleaving reduces back-reflection, a portion of the reflected pump still couples into the detection stage at a power level comparable to that of the probe reaching the photodetector, leading to a noisy DC background in detection. To improve the Brillouin measurement contrast and enhance SNR, a Brillouin depletion system is added to suppress the unwanted reflected pump before photodetection. This is achieved by launching the light from a distributed feedback (DFB) laser ( ~ 1 MHz linewidth) into a 730 m SMF spool, counterpropagating with the reflected pump. By tuning the frequency difference between the two signals to match the BFS of the SMF, the reflected pump is attenuated by over 25 dB, while the probe signal (several hundred MHz away and carrying the gas SBS information) remains unaffected. This ensures that at the input of the photodetector, the residual pump reflection remains >25 dB below the probe signal of interest, significantly improving the detection quality.

Brillouin responses are measured at discrete temperature steps. Although a short waiting period is introduced after each change, the gas-filled HCF actually reaches thermal equilibrium within only a few milliseconds, as calculated in Supplementary Information 5.1. For temperature reference, a commercial platinum PT-1000 thermo probe has been placed in close contact with the fibre under test. This device has been previously calibrated in the lab at two fixed points: boiling water (371.820 K at 96 700 Pa) and liquid nitrogen (76.961 K at 96 700 Pa), providing a temperature accuracy of ~0.26 mK. However, the main objective of the work is to characterize gas-based Brillouin thermometry and demonstrate its feasibility, particularly at cryogenic temperatures, rather than to optimize the system to its ultimate precision. Consequently, the temperature steps are not applied comparable to the resolution. From this perspective, there is clear potential to further improve the sensing performance by implementing conventional strategies widely established in the fibre-optic sensing field.

### Experimental setup for gas-based distributed Brillouin temperature sensing

**Fig. 12:**
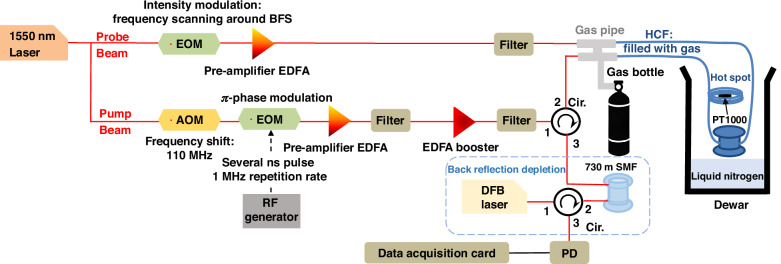
Experimental setup for distributed thermometry in gases at cryogenic temperatures. This time-domain setup is based on the Brillouin Echoes distributed sensing technique, enabling high-resolution temperature measurements along a gas-filled HCF. The method achieves spatial resolutions significantly shorter than those defined by the acoustic lifetime in gases, allowing for distributed sensing with centimetre-scale resolution over a 18.5 m-long fibre. The system is designed to operate across a wide temperature range, including cryogenic conditions

Figure 12 illustrates the experimental setup used for gas-based Brillouin distributed temperature sensing, which is based on a Brillouin echoes configuration^43^. In this approach, a dual-sideband probe with suppressed carrier counter-propagates with a continuous-wave pump, which is periodically modulated with a $\pi$-phase shifted pulse for several nanoseconds, with a repetition period of 1 μs. Due to the small BFS of the analysed gases and the impracticality of using an extremely narrow optical filter to select a single probe sideband, the pump optical frequency is upshifted by an AOM. The pump and probe optical frequencies are then adjusted to secure that the low-frequency probe sideband interacts with the pump, enabling efficient Brillouin gain interaction. Given the long pulse repetition period, being much longer than the acoustic response time constant of about 30 ns in the analysed gases, the Brillouin acoustic wave can be efficiently activated in the gas along the entire HCF length. During the short $\pi$-phase shifted pulse, which last significantly shorter than acoustic response time constant, the amplitude and phase of the acoustic wave remains nearly unchanged, reflecting the pump completely out of phase compared to the steady-state gain situation. This destructive interference leads to a small, local apparent loss on the probe wave, providing a high spatial resolution of tens of centimetres while preserving the natural narrow Brillouin spectral width^43^.

To create a longitudinal temperature variation in the gas-filled HCF, the first metres of the fibre are positioned several centimetres above the main HCF spool (which contains the longest fibre section) inside the dewar. This elevated short section of fibre acts as a hot spot, maintaining a higher temperature than the main spool, which remains at a lower and therefore colder position.

## Supplementary information

Supplementary Information for Absolute thermometry based on Brillouin scattering in gases

## Data Availability

Data underlying the results presented in this paper are available from the authors upon reasonable request.
