# Supplementary material for: Absolute thermometry based on Brillouin scattering in gases
Source: Light Sci Appl. 2026 Jan 12;15:69. doi: 10.1038/s41377-025-02168-3 (PMC12791128; doi:10.1038/s41377-025-02168-3)
Supplement: Supplementary file 1 — Supplementary Information for Absolute thermometry based on Brillouin scattering in gases [file 41377_2025_2168_MOESM1_ESM.docx]

## SUPPLEMENTARY INFORMATION FOR

Absolute thermometry based on Brillouin scattering in gases

Yuting Yang^1^, Marcelo A. Soto^2^ and Luc Thévenaz*^1^

^1^EPFL Ecole Polytechnique Fédérale de Lausanne, Institute of Electrical and Micro Engineering, Station 11, 1015 Lausanne, Switzerland;

^2^Department of Electronics Engineering, Universidad Técnica Federico Santa María,
2390123 Valparaiso, Chile

Official email addresses:

Yuting Yang [yuting.yang@epfl.ch](mailto:yuting.yang@epfl.ch)

Marcelo A. Soto [marcelo.sotoh@usm.cl](mailto:marcelo.sotoh@usm.cl)

Luc Thévenaz [luc.thevenaz@epfl.ch](mailto:luc.thevenaz@epfl.ch)

Full contact details of the corresponding author (including telephone number):

Luc Thévenaz [luc.thevenaz@epfl.ch](mailto:luc.thevenaz@epfl.ch) +41 21 693 47 74

**Supplementary Information 1: Study of thermodynamic behaviour of gases**

This section provides a general overview of fundamental gas properties, with a focus on the thermodynamic behaviour of both ideal and non-ideal gases. The objective is to introduce the key principles and concepts needed to derive the acoustic properties of gases, such as the sound velocity and losses, which are essential for developing explicit models of the Brillouin frequency shift (BFS), as discussed in the main text of the paper, as well as the Brillouin spectral width and gain coefficient described in Supplementary Information 2.

An ideal gas is a theoretical concept in physics and chemistry used to describe the behaviour of gases under certain simplified assumptions. In this model, gas molecules follow these principles^1^:

- They are treated as point particles with no volume.
- Collisions between molecules are perfectly elastic, with no loss of energy, and no intermolecular forces of attraction or repulsion.

The corresponding equation of state for an ideal gas is expressed as^1^:

$PV=mRT$ (S1)

where $m$ is the amount of substance (number of moles) of the gas, $P$ is the gas pressure, $R=8.3145 J\cdot K^{-1}\cdot\mathrm{mol}^{-1}$ is the universal gas constant, and $T$ is the absolute temperature.

Many real gases, such as nitrogen, oxygen, hydrogen, noble gases, and air, closely follow the ideal gas behaviour under specific conditions. This approximation is mostly accurate at high temperatures and low pressures, where intermolecular forces and molecular size are negligible compared to the kinetic energy of the particles and the space between them^1^.

However, at low temperatures or high pressures, the ideal gas law becomes less accurate due to the influence of intermolecular forces and the finite volume of gas molecules. To account for these factors, the van der Waals model^2^ is introduced here as a refinement. The equation of state for a van der Waals gas is described as^2^:

$\left( P+\frac{m^{2}a}{V^{2}} \right)(V-mb)=mRT$ (S2)

where $a$ and $b$ are gas-dependent parameters known as van der Waals constants, which account for intermolecular forces and molecular volume, respectively.

In the context of Brillouin interaction within the van der Waals model, key quantities —such as the acoustic velocity— are more straightforward when formulated in terms of the gas density * *. An explicit analytical expression for * *can be algebraically derived from the van der Waals equation of state $(PM_{m}^{2}+a\rho^{2})(M_{m}-b\rho)=\rho RTM_{m}^{2}$ (obtained from Supplementary Eq. (S2), where $M_{m}$ is the molar mass) as follows:

$$\frac{\rho}{M_{m}}= \sqrt[3]{\left( \frac{1}{27b^{3}}-\frac{RT}{6ab^{2}}+\frac{P}{3ab} \right)+\sqrt{\left( -\frac{1}{27b^{3}}+\frac{RT}{6ab^{2}}-\frac{P}{3ab} \right)^{2}+\left( \frac{1}{3ab}(RT+Pb)-\frac{1}{9b^{2}} \right)^{3}}}$$

$$+\sqrt[3]{\left( \frac{1}{27b^{3}}-\frac{RT}{6ab^{2}}+\frac{P}{3ab} \right)-\sqrt{\left( -\frac{1}{27b^{3}}+\frac{RT}{6ab^{2}}-\frac{P}{3ab} \right)^{2}+\left( \frac{1}{3ab}(RT+Pb)-\frac{1}{9b^{2}} \right)^{3}}} (S3)$$

This formulation provides a closed-form expression in which the dependence of key quantities on temperature *T* is fully explicit. However, as seen in Supplementary Eq. (S3), a residual dependence on the gas pressure *P* remains inherent to the van der Waals model.

- 1. **Adiabatic process**

In our Brillouin study, we focus on the acoustic behaviour of real gases, relying primarily on the classical ideal gas model^2^, which provides sufficient precision for the analysis. In the context of Brillouin scattering for thermometry, we assume that the acoustic wave propagation occurs much faster than the heat conduction, allowing us to treat the gas molecules as if they were confined in a thermally isolated container. This scenario describes an adiabatic process^3^, where no heat is exchanged with the external surroundings. For an ideal gas, such adiabatic process obeys to the condition^1^:

$PV^{\gamma}=\mathrm{constant}$ (S4)

where $\gamma$ is the heat capacity ratio, defined as the ratio between $C_{P}$, the specific heat at constant pressure $P$, and $C_{V}$, the specific heat at constant volume $V$.

For a real gas described by van der Waals model, the adiabatic process obeys to the modified condition^2^:

$\left( P+\frac{m^{2}a}{V^{2}} \right){(V-mb)}^{\gamma}=\mathrm{constant}$ (S5)

These adiabatic relationships are essential for deriving explicit expressions for the acoustic velocity and BFS in gases, as described in Eqs. (3) and (4) of the main text of this paper, for the ideal and non-ideal cases, respectively.

Under the approximation that the acoustic wave propagation is much faster than the heat conduction, the acoustic process can be treated as adiabatic and reversible. In this case, the adiabatic bulk modulus $\kappa_{s}$ is calculated as^4^: $\kappa_{s}=-V\left( \frac{\partial P}{\partial V} \right)_{s}$, where *P* is the gas pressure, *V* is the gas volume, and the derivative is taken at constant entropy *S*. For an ideal gas, using Supplementary Eq. (S4), this simplifies to $\kappa_{s}=-V\left( \frac{\partial P}{\partial V} \right)_{s}=\gamma P$.

- 1. **Basic thermodynamic properties**

Based on the definition of specific heat, $C_{V}={\partial U}/{\partial T}$ describes how the internal energy $U$ of a gas changes with temperature $T$ while keeping the volume $V$ constant. Meanwhile, $C_{P}{=\partial H}/{\partial T}$ evaluates the changes of the enthalpy $H$ with respect to temperature $T$ at constant pressure $P$​. Under the ideal gas approximation, $C_{V}=3/2R$ and $C_{P}=5/2R$ for monatomic gases, while $C_{V}=5/2R$ and $C_{P}=7/2R$ for diatomic gases^1^, where $R=8.3145 J\cdot K^{-1}\cdot{mol}^{-1}$ is the universal gas constant. This results in a heat capacity ratio $\gamma=5/3$ or $\gamma=7/5$, for monatomic or diatomic gases, respectively.

In the case of a van der Waals gas, the internal energy $U$ consists of two parts^5^: $U_{ideal}$, the internal energy of an ideal gas, and $U_{attraction}\propto{-ma}/V$, an additional term that accounts for intermolecular attractions in the van der Waals model. However, because the volume $V$ is typically large compared to the molecular size in most practical scenarios, $U_{attraction}$ becomes negligible^5^. As a result, $C_{V}$ can be approximated by the same value as in the ideal gas model, i.e. $C_{V}=3/2R$ and $C_{V}=5/2R$, for monatomic and diatomic gases, respectively. Similarly, the enthalpy $H=U+PV$, also deviates slightly from its ideal counterpart $H_{ideal}=U_{ideal}+P_{ideal}V_{ideal}$. Since $U\approx U_{ideal}$, the deviation of enthalpy $\Delta H$ can be described as: $\Delta H\approx PV-P_{ideal}V_{ideal}=Pb+{m^{2}ab}/{V^{2}}-{ma}/V$. Note that this deviation is small under our experimental conditions, where the pressure is not particularly high, and the volume is relatively large. As a result, $C_{P}$ approximates $C_{P\_ideal}$, being $7/2R$ and $7/2R$ for monatomic and diatomic gases.

In conclusion, the specific heats $C_{P}$ and $C_{V}$ of a van der Waals gas can be treated analogously to those of an ideal gas, leading to $\gamma=5/3$ for monatomic gases and $\gamma=7/5$ for diatomic gases. These properties and values are used in this work to derive explicit expressions for the BFS presented in the main text, as well as for the Brillouin spectral width and gain coefficient, detailed in Supplementary Information 2.

- 1. **Fluid properties**
     1. Viscosity

Gas viscosity plays a key role in determining the acoustic loss (or attenuation) in a gases, as it governs how a gas resists sound wave propagation by mediating the energy transfer between molecules. Accurate modelling of the gas viscosity is therefore essential for deriving mathematical expressions for the Brillouin linewidth and gain, both of which are directly determined by the acoustic losses in the gas. The viscosity $\eta$ of a gas arises from molecular momentum transfer and depends on the gas temperature and molecular interactions.

In the context of real gases, there are two main types of viscosity: *i)* the shear viscosity $\eta_{s}$, which accounts for internal friction due to the relative motion of adjacent gas layers that results in energy dissipation, and *ii)* bulk viscosity $\eta_{b}$, which captures the energy dissipation during uniform gas compression or expansion.

For an ideal gas, where molecules do not experience intermolecular forces, momentum transfer still occurs through elastic molecular collisions, leading to shear viscosity and energy dissipation in gas layers. However, since energy is conserved during volume changes in ideal gases, $\eta_{b}=0$.

In contrast, for real (non-ideal) gases, $\eta_{b}$ is generally nonzero due to additional energy dissipation mechanisms. It is often estimated as a fraction of $\eta_{s}$, expressed as^6^: $\eta_{b}={c_{\eta}\eta}_{s}$, where $c_{\eta}$ is a proportionality constant that depends on the gas type. For inert (monoatomic) gases $c_{\eta}=0$, while for diatomic gases^6^ $c_{\eta}\approx0.67$.

Under the widely used mean free path collision model in thermodynamic studies^7^, the viscosity can be approximated as:

$\eta^{(mfp)}=\frac{1}{3}\rho\lambda_{m}\bar{\upsilon}_{m}$ (S6)

where$\rho=PM_{m}/RT$ is the gas density, $M_{m}$ is the molar mass of the gas molecules, $\lambda_{m}$ is the mean free path of the molecules and $\bar{\upsilon}_{m}$ is the average molecular velocity, which can be derived using kinetic theory as^7^:

$\lambda_{m}=\frac{k_{B}T}{\sqrt{2}\pi d^{2}P}$, (S7)

and $\bar{\upsilon}_{m}=\sqrt{\frac{8RT}{\pi M_{m}}}$ (S8)

where $d$ represents the gas molecular diameter and $k_{B}=1.380649\times{10}^{-23} J\cdot K^{-1}$ is the Boltzmann constant. The relationship between the universal gas constant $R$ and $k_{B}$ is described as: $R=N_{A}k_{B}$, where $N_{A}=6.022\times{10}^{23}\mathrm{mol}^{-1}$ is the Avogadro's number, which represents the number of constituent particles (molecules in our case) in one mole of a substance

Note that the kinetic diameter of a gas molecule is closely related to its mean free path, which represents the average distance that a particle travels before colliding with another. For a fast-moving particle (that is, one that moves significantly faster than the surrounding particles, the kinetic diameter is defined as^7^: $\lambda_{m}=\frac{1}{\pi d^{2}n_{m}}$, where $n_{m}=\frac{P}{k_{B}T}$ is the number density of gas molecules. In a more common scenario, where the particle of interest is indistinguishable from the surrounding gas molecules, the Maxwell–Boltzmann distribution of energies must be taken into account^7^. This leads to a mean free path: $\lambda_{m}=\frac{1}{\sqrt{2}\pi d^{2}n_{m}}$=$\frac{k_{B}T}{\sqrt{2}\pi d^{2}P}$. The factor $1/3$ in Supplementary Eq. (S6) arises from the assumption that molecules reaching a plane have, on average, undergone their last collision at a distance of approximately $2/3$ of the mean free path, leaving $1/3$ for the collision^7^. As a result, the shear viscosity described in Supplementary Eq. (S6) is estimated as:

$\eta_{s}^{(mfp)}=\sqrt{\frac{4RM_{m}T}{9N_{A}^{2}\pi^{3}d^{4}}}$ (S9)

However, it is important to note that the mean free path collision model introduces non-negligible estimations errors when calculating $\eta_{s}$. To improve the accuracy of the prediction, we adopted the well-established Chapman–Enskog theory^8^, which provides a more refined calculation of the dynamic viscosity by incorporating a more detailed collision model. According to this theory, the shear viscosity can be estimated as^8^:

$\eta_{s}=\frac{5}{16d^{2}}\sqrt{\frac{RM_{m}T}{N_{A}\pi}}\frac{1}{1+\frac{S}{T}}$, (S10)

where $d$ is the hard-sphere diameter of the gas molecules, and $S$ is a constant that depends on the gas species (e.g. $S= 50 K$ for neon, $S=107 K$ for nitrogen and $S=114$ K for argon^8^). In Supplementary Information 2, we will compare the Brillouin linewidth calculations using the viscosity values obtained from Supplementary Eqs. (S9) and (S10), demonstrating the improved precision of the shear viscosity estimation provided by Supplementary Eq. (S10).

- - 1. Thermal conductivity

It is important to notice that heat transfer in a gas is mediated by thermally activated acoustic phonons^9^, so when analysing the energy transfer by acoustic phonons, the thermal conductivity must be considered, as it plays a crucial role in determining how efficiently heat is transferred within the medium. In general, can be expressed as^10^:

$\kappa=\frac{C_{p}}{M_{m}P_{r}}\eta_{s}$ (S11)

where $P_{r}=\frac{4}{9-5}$ is the Prandtl number^10^, a dimensionless constant that depends on the heat capacity ratio $\gamma$ of different gas species. Under the ideal gas model, as previously mentioned, $\gamma=5/3$ for monatomic gases and $\gamma=7/5$ for diatomic gases, resulting in $P_{r}=2/3$ and $P_{r}=14/19$, respectively. This formulation is derived from the Eucken equation^11^, which provides a theoretical framework for estimating the thermal conductivity of gases. Using this formulation and Supplementary Eq. (S11), the acoustic loss associated with Brillouin scattering is calculated in Supplementary Information 2.

**Supplementary Information 2: Brillouin linewidth and gain coefficient study**

In this section, we derive mathematical models for the Brillouin spectral width and gain coefficient as functions of the fundamental gas properties described in Supplementary Information 1, for both ideal and non-ideal gases. While the Brillouin frequency shift (BFS)—linked to the acoustic velocity—is the key parameter enabling absolute temperature measurements and is discussed in the main text, our focus here is on modelling the Brillouin linewidth and gain. These parameters are essential for providing a complete and comprehensive description of the Brillouin spectral response in gases, which is critical for thermometry applications. As noted in Eq. (5) of the main text, the linewidth and gain strongly influence the temperature resolution and overall performance of Brillouin-based temperature sensors.

- 1. **Brillouin linewidth prediction**

The Brillouin linewidth is fundamentally determined by the fluid properties of gas medium directly depending on thermodynamic quantities, and is expressed as^12^:

$\Delta\nu_{B}=\frac{q^{2}}{2\pi\rho}\left[ \frac{4}{3}\eta_{s}+\eta_{b}+\frac{\kappa M_{m}}{C_{p}}\left( \gamma-1 \right) \right]$ (S12)

where $q \approx2n\omega/c$ is the wavenumber for backward SBS, ${}_{s}$ and ${}_{b}$ are the shear and bulk viscosities, is the thermal conductivity, and $\rho$ is the gas density. Note that this equation highlights how the Brillouin linewidth is directly influenced by the key fluid properties of the gas medium, allowing it to be predicted based on the gas models discussed in Supplementary Information 1.

For an ideal gas, where ${}_{b}= 0$ and the thermal conductivity is given by $\kappa=\frac{C_{p}}{M_{m}P_{r}}\eta_{s}$, with $P_{r}=2/3$ for monatomic gases and $P_{r}=14/19$  for diatomic gases, the Brillouin linewidth simplifies to:

$\Delta\nu_{B}^{\left( ideal \right)}\left( T,P \right)=\frac{4RT\eta_{s}}{{\lambda_{0}}^{2}M_{m}P}\left( \frac{4}{3}+\frac{\gamma-1}{P_{r}} \right)$ (S13)

On the other hand, for a (non-ideal) van der Waals gas, the bulk viscosity follows$\eta_{b}=c_{\eta}\eta_{s}$, where $c_{\eta}$ is a proportionality constant that depends on the gas type^6^ ($c_{\eta}=0$ for inert gases, and $c_{\eta}\approx0.67$ for the diatomic gases^6^ used in our experiments). The thermal conductivity remains the same as in the ideal gas model, leading to the following expression for the Brillouin linewidth:

$\Delta\nu_{B}^{\left( vdW \right)}\left( T,P \right)=\frac{q^{2}\eta_{s}}{2\pi\rho}\left( \frac{4}{3}+c_{\eta}+\frac{\gamma-1}{P_{r}} \right)$ (S14)

At this stage, the only remaining parameter to be determined is the shear viscosity $\eta_{s}$. As discussed in Supplementary Section 1.3, there are two primary models for estimating the shear viscosity: the commonly used mean free path collision model^7^ and the more accurate Chapman–Enskog theory^8^. To evaluate the impact of these models on the Brillouin linewidth predictions, a comparative analysis is conducted using three different viscosity estimation methods: *i)* the mean free path collision model^7^, *ii)* the Chapman–Enskog theory^8^, and *iii)* empirical data from the NIST webbook^13^. The resulting Brillouin linewidth calculations based on these three approaches are presented in Fig. S1, where the blue curve represents the SBS linewidth predicted using the mean free path model, the black curve corresponds to predictions based on the Chapman-Enskog model, and the red curve (practically under the black curve) represents the linewidth obtained from the NIST empirical data^13^. To further validate these predictions, the theoretical linewidths as a function of temperature are compared with experimental measurements (represented by red dots) for neon gas at 10 bar (note that the same pressure is used in the theoretical calculations). Results demonstrate an excellent agreement between the experimental data and the linewidth predictions based on the Chapman-Enskog model, confirming its higher accuracy in describing the shear viscosity in this context.

**Fig. S1.** Comparison of measured Brillouin linewidths (red dots) with theoretical predictions for **a** 10 bar Ne and **b** 10 bar Ar. Three approaches are considered: the mean free path collision model (blue line), the Chapman–Enskog theory (black line), and viscosity-based calculations using NIST Webbook data (red line). Among these, the Chapman–Enskog model shows the best agreement with experimental measurements.

In conclusion, to achieve more accurate Brillouin linewidth predictions, we adopt the Chapman–Enskog theory instead of the mean free path collision model for the shear viscosity estimation. This approach provides a more precise characterisation of the relationship between shear viscosity ($\eta_{s}$​) and temperature ($T$), ensuring improved reliability in our analysis. Accordingly, all viscosity-related calculations presented in the work are based on the Chapman–Enskog theory for consistency and accuracy.

- 1. **Brillouin gain coefficient prediction**

In general, the Brillouin gain coefficient is expressed as^14^:

$\gamma_{B}=\frac{\gamma_{e}^{2}\omega^{2}}{\rho nV_{a}c^{3}2\pi\Delta\nu_{B}A_{eff}^{ao}}$ (S15)

where *c* is the speed of light in vacuum, $A_{eff}^{ao}$ is the acousto-optic overlap effective area in the waveguide (80 μm^2^ for our HCF), $\omega$ is the light angular frequency and $\gamma_{e}$ is the electrostrictive constant in the gas medium, which can be calculated as^15^ $\gamma_{e}=(n^{2}-1)(n^{2}+2)/3$.

Using the thermal properties discussed in Supplementary Information 2.1, the Brillouin gain coefficient under ideal gas model can be calculated as:

$\gamma_{B}^{\left( ideal \right)}\left( T,P \right)=\frac{{k_{DG}}^{2}{M_{m}}^{2.5}P^{2}{(k_{DG}\frac{PM_{m}}{RT}+2)}^{2}}{4cA_{eff}^{ao}\cdot\eta_{s}\left[ 4/3+\left( \gamma-1 \right)/{P_{r}} \right]\sqrt{\gamma}\left( k_{DG}\frac{PM_{m}}{RT}+1 \right)^{3}R^{2.5}T^{2.5}}$ (S16)

where the electrostrictive constant for an ideal gas is applied as^15^ $\gamma_{e}^{\left( ideal \right)}=(n^{2}-1)(n^{2}+2)/3\approx k_{DG}\frac{PM_{m}}{RT}\left( k_{DG}\frac{PM_{m}}{RT}+2 \right)$.

Similarly, for a (non-ideal) van der Waals gas, the Brillouin gain coefficient is given as:

$\gamma_{B}^{\left( vdW \right)}\left( T,P \right)=\frac{{k_{DG}}^{2}\rho^{2}\left( k_{DG}\rho+2 \right)^{2}}{4cA_{eff}^{ao}\eta_{s}\left( \frac{4}{3}+c_{\eta}+\frac{\gamma-1}{P_{r}} \right)\left( k_{DG}\rho+1 \right)^{3}\sqrt{\frac{\gamma}{\rho}\frac{M_{m}}{M_{m}-b\rho}\left( P+\frac{a\rho^{2}}{M_{m}^{2}} \right)-\frac{2a\rho}{M_{m}^{2}}}}$ (S17)

where the shear viscosity $\eta_{s}$ is estimated using the Chapman–Enskog theory for better accuracy.

- 1. **Brillouin gain coefficient measurement**

Note that a dual-intensity modulation scheme^14^ is applied to both pump and probe waves for selectively measuring the nonlinear Brillouin response during the experiments. In this setup, the detected electrical Brillouin signal is determined not only by the pump and probe powers but also by the used modulation frequency and depth. The Brillouin gain coefficient is then determined from the measurements obtained using a lock-in amplifier, following the expression provided in the literature^14^, which takes into account the nonlinear response of the electro-optic modulators:

$\gamma_{B}=\frac{\left( 1-J_{0}(2\varsigma_{P}) \right)\left( 1-J_{0}(2\varsigma_{S}) \right)V_{s, det}}{2J_{2}(2\varsigma_{P})J_{2}(2\varsigma_{P})\rho_{PD}P_{s, det}P_{P0}L_{eff}\alpha_{F}\zeta}$ (S18)

where $J_{i}$ represents the $i$-th order Bessel function of the first kind, $\varsigma_{P}$ and $\varsigma_{S}$ are the modulation depths applied to the pump and probe waves before interaction, $V_{s, det}$ is the lock-in amplifier voltage that is proportional to the amplitude of the Brillouin gain at the specific scanned pump-probe offset frequency, $\rho_{PD}$ is the photodetector power-to-voltage conversion factor, $P_{s, det}$ is the optical probe power at the photodetector, $P_{P0}$ is the optical pump power at the input of the HCF, $L_{eff}$ is the fiber effective length, $\alpha_{F}$ accounts for any voltage attenuation caused by electrical bandpass filters at the reception stage, and $\zeta$ is a constant that characterises the setup’s response at a given modulation frequency^14^. This formulation ensures that the Brillouin gain coefficient is accurately extracted from the lock-in measurements, while accounting for all relevant system parameters.

**Supplementary Information 3: BFS in conventional silica single-mode fibres**

It is important to note that Brillouin-based cryogenic temperature sensing in solid-core silica fibres faces significant limitations and problems beyond the need for pre-calibration. As shown in Fig. S2, the BFS response becomes non-monotonic at temperatures below approximately 120 K, and even its temperature sensitivity (represented by the slope of the BFS response) vanishes around 80 K, introducing potential ambiguities and compromising measurement reliability in this temperature range^16,17^.

**Fig. S2.** Brillouin frequency shift as a function of temperature for a conventional silica SMF (raw data reused from^17^with permission). The curve demonstrates a significantly reduced temperature sensitivity—evident from the shallow slope—in the cryogenic temperature range, highlighting the limitations of silica-based Brillouin sensing at low temperatures.

The BFS temperature sensitivity is actually a critical parameter, as it defines how effectively BFS variations can be translated into temperature changes. In silica fibres, this sensitivity decreases significantly below ~120 K, and the BFS becomes non-monotonic, making reliable Brillouin-based temperature sensing in this range nearly impossible. Other scattering-based techniques in silica fibres, such as Raman and Rayleigh scattering, also suffer from reduced sensitivity at cryogenic temperatures. In contrast, Brillouin scattering in gases offers an enhanced and monotonic response in the cryogenic range, enabling more accurate and reliable temperature measurements under these conditions.

**Supplementary Information 4: BFS sensitivity in gas**

Using the ideal gas model, the BFS sensitivity is pressure-independent and can be calculated by taking the temperature derivative of Eq. (3) from the main text, as follows:

$\frac{d\nu_{B}^{\left( ideal \right)}}{dT}=\frac{1}{\lambda_{0}}\left( \frac{k_{DG}PM_{m}}{RT}+1 \right)\sqrt{\frac{\gamma R}{M_{m}T}}$ (S19)

However, when using the van der Waals model, the BFS sensitivity can be obtained by differentiating Eq. (4) from the main text with respect to temperature, as follows:

$$\frac{\partial\nu_{B}^{\left( vdW \right)}}{\partial T}=\frac{\partial\left[ \frac{2\left( k_{DG}\rho+1 \right)}{\lambda_{0}}\sqrt{\frac{\gamma}{\rho}\frac{M_{m}}{M_{m}-b\rho}\left( P+\frac{a\rho^{2}}{{M_{m}}^{2}} \right)-\frac{2a\rho}{{M_{m}}^{2}}} \right]}{\partial T}$$

$=\frac{\partial\rho}{\partial T}\left\{ \frac{2k_{DG}}{\lambda_{0}}\sqrt{\frac{\gamma}{\rho}\frac{M_{m}}{M_{m}-b\rho}\left( P+\frac{a\rho^{2}}{{M_{m}}^{2}} \right)-\frac{2a\rho}{{M_{m}}^{2}}}+\frac{k_{DG}\rho+1}{\lambda_{0}\sqrt{\frac{\gamma}{\rho} \frac{M_{m}}{M_{m}-b\rho}\left( P+\frac{a\rho^{2}}{{M_{m}}^{2}} \right)-\frac{2a\rho}{{M_{m}}^{2}}}}\left\{ \frac{-2a}{{M_{m}}^{2}}+\left( P+\frac{a\rho^{2}}{{M_{m}}^{2}} \right)\left[ \frac{\gamma}{\rho}\frac{bM_{m}}{\left( M_{m}-b\rho\right)^{2}}-\frac{\gamma}{\rho^{2}}\frac{M_{m}}{M_{m}-b\rho} \right]+\frac{\gamma}{\rho}\frac{M_{m}}{M_{m}-b\rho}\frac{2a\rho}{{M_{m}}^{2}} \right\} \right\}$ (S20)

where $\frac{\partial\rho}{\partial T}$ can be obtained based on the calculation of $\rho$ as the only real root of the third-degree polynomial expression described in Supplementary Eq. (S6), derived from the van der Waals state equation.

**Fig. S3.** Comparison between experimentally measured BFS temperature sensitivity (dots) and theoretical predictions based on gas models (solid lines) for **a** 10 bar Ne and **b** 0.5 bar N₂. The results show good agreement across the temperature range, validating the accuracy of the gas-based models.

To validate the relationship between the BFS temperature sensitivity and temperature, BFS measurements are carried out on 10 bar Ne and 0.5 bar N_2_ and the numerical derivative of the measured BFS values with respect to temperature is computed. Fig. S3 compares these numerical results (dots) with the theoretical predictions from gas models (solid lines), showing very good agreement.

**Supplementary Information 5: Complementary information on Brillouin scattering experiments in gases**

1. 1. **Inducing temperature changes in cryogenic conditions**

In this work, Brillouin measurements are carried out using a HCF filled with gases at controlled pressures, continuously monitored by a precision pressure gauge. To maintain a stable gas density, the system is connected to a large gas tank, ensuring a constant gas supply throughout the experiments. The main section of the HCF (18.5 m) is exposed to controlled temperature variations to characterise the Brillouin spectral response of the gas as a function of temperature. For temperatures near ambient conditions (273.15 K to 343.15 K), the HCF spool is immersed in a water bath with a precise temperature control. For cryogenic measurements, a dewar with liquid nitrogen (boiling point of 77 K at 1 atmospheric pressure) provides a low-temperature environment with varying temperatures, allowing Brillouin scattering to be characterised down to 77 K. To achieve different temperature variations, the 2 cm-thick HCF spool, along with a PT1000 thermal probe for reference, is either placed inside or above the liquid nitrogen. By adjusting the height of the spool, the system enables gradual temperature changes. To reach temperatures below 77 K, evaporative cooling is employed: a constant vacuum is applied inside the sealed dewar, accelerating the evaporation of the liquid nitrogen and removing latent heat from the surface, thus allowing the system to reach a minimum temperature of 67 K. However, it must be noted that when the HCF spool is positioned above the liquid nitrogen surface, vertical temperature gradients can introduce inhomogeneities. These gradients vary depending on the nitrogen fill level and can lead to the small systematic errors observed around 150 K in Fig. 4 and Fig. 5 of the main text.

- 1. **Gas filled spliced HCF scheme**

The current gas-based Brillouin scattering system, as described in the main text, involves external gas pipelines and bottles, which presents practical limitations for real-world applications. To simplify the system, we propose a method using pre-filled HCF with both ends spliced, ensuring light guidance while remaining gas tight. The proposed procedure can be performed in a laboratory environment or a small production line. First, a particular section of HCF is cooled down, for example by immersion in liquid nitrogen, while both open ends remain connected to gas lines to allow gas flow through the fibre. This cooling leads to the formation of a localized reservoir of condensed gas molecules. After thermal stabilisation is achieved, the HCF ends are disconnected from the gas supply and quickly spliced to SMFs, the automatic evaporation from the cooled section creating a gas push to minimise the contamination from the ambient air into the fiber. To reduce gas loss during this process, the cooled section is kept at cryogenic temperatures until splicing is completed.

Another approach to further reduce the gas loss during the fibre splicing process involves first splicing one end of the HCF while keeping the opposite end free end connected to the gas supply pipeline. A cold gas reservoir is then created by condensing the gas within the fibre, as illustrated in Fig. S4a. Once the cold reservoir is established and the gas is stabilised within the fibre, the remaining free end is rapidly spliced to seal the system, as shown in Fig. S4b. The main limitation of this method lies in the reduced gas flow efficiency during the condensation stage, as only one end of the HCF is open to the gas source. A previous study on gas flow in HCFs^18^ has already demonstrated the feasibility of preparing this pre-filled and spliced HCF configuration.

**Fig. S4.** Two-step procedure for preparing a gas-filled, fully spliced HCF with minimal gas loss. **a** In the first step, one end of the HCF is pre-spliced and the other end remains open, allowing the fibre to be filled with gas. A condensed gas reservoir is formed by cooling down a section of the HCF, stabilising the internal gas. **b** In the second step, after gas stabilisation, the remaining free end is rapidly spliced to seal the system. This method reduces gas leakage during the final splicing process.

When the gas used is sufficiently diluted and far from its liquefaction point, it can be approximated as an ideal gas. In this regime, changes in the gas density have a negligible effect on the refractive index, which can be approximated as 1. For instance, in the case of neon, the refractive index deviation is less than 0.01% per bar (e.g. for neon, calculated based on Dale-Gladstone relation^19^), so that the BFS follows the relation: $\nu_{B}=\frac{2}{\lambda_{0}}\sqrt{\frac{\gamma RT}{M_{m}}}$, which is independent of pressure and directly correlates with temperature. This makes the approach particularly suited for distributed temperature sensing, with the only drawback being the relatively weak SBS signal resulting from the low gas density.

In contrast, when using a highly condensed gas to enhance the Brillouin gain, gas density variations become non-negligible. Unlike the open-ended configuration used in the current study, a sealed fibre acts as a isolated system with a fixed number of gas molecules. In distributed sensing applications, the local gas density is not solely determined by the local temperature but is also influenced by the overall temperature distribution along the fibre. This is because colder sections of the fibre experience an increase in local gas density due to condensation, while warmer sections see a decrease in density as gas molecules migrate toward the cold regions. These spatial variations in density affect both the refractive index and the adiabatic bulk modulus $\kappa_{s}$, and consequently alter the BFS, as described in Eq. (4) of the main text. Accurate temperature retrieval in such sealed systems requires taking into account these spatial density changes. This can be achieved through a correction algorithm based on the conservation of the total number of gas molecules in the HCF. By incorporating the fibre’s volume distribution across different temperature zones and their corresponding temperatures, it is possible to estimate the local densities and refine the BFS–temperature relationship through an iterative and converging calculation loop.

This complex behaviour in sealed systems presents an intriguing opportunity for future research. The interplay between temperature gradients, molecular migration, and resulting density variations creates a spatially dynamic environment in which the refractive index, adiabatic bulk modulus $\kappa_{s}$, and ultimately the Brillouin frequency shift are all interdependent. Understanding and accurately modelling this coupled behaviour could enable advanced temperature mapping techniques in closed gas-filled fibres. Developing reliable calibration and compensation methods for such systems—especially under strong thermal gradients—represents a promising and challenging avenue for further investigation. However, this analysis lies beyond the scope of the current work and is proposed as a subject for future studies.

**Supplementary Information 6: Fitting of the Brillouin spectrum measurement**

The experimental setup in Fig. 11 of the main text enables the simultaneous measurement of both the Brillouin gain spectrum at $f_{scan}={}_{B}-f_{AOM}$ and the Brillouin loss spectrum at $f_{scan}={}_{B}+f_{AOM}$, provided that $f_{scan}$covers a sufficiently wide spectral range. Figs. S5a and S5b show the magnitude of the simultaneous Brillouin gain and loss spectral measurements obtained by lock-in detection for narrow and broad Brillouin linewidth conditions, respectively. The Brillouin linewidth and BFS are extracted by performing a curve fitting on the entire spectrum using the absolute value of the sum of two Lorentzian distributions with opposite signs, representing Brillouin gain (+) and loss (-) interactions. Since the measurements rely on the amplitude of the signal obtained from the lock-in detection, the calculation of the absolute value is required for accurate fitting. These two Lorentzian distributions share the same linewidth and are symmetrically positioned around the BFS, separated by $f_{AOM}$. For narrow Brillouin linewidths, the gain and loss spectra remain well separated, as clearly shown in Fig. S5a, providing an accuracy comparable to the traditional single-spectrum Lorentzian fitting of either the SBS gain or loss, but with a slight improvement due to the dual-peak fitting approach. However, when the Brillouin linewidth broadens to a value comparable to $f_{AOM}$, the gain and loss spectra start to overlap, producing a sharp transition around the BFS, as depicted in Fig. S5b. Thanks to the use of two Lorentzian functions, this enhanced fitting method provides a robust approach for accurately determining the peak positions of both spectra, significantly enhancing the BFS and linewidth accuracy compared to a traditional single-peak Lorentzian fitting method. This way, this technique enables highly precise measurements, particularly in low-gain conditions, such as at high temperatures or low gas pressures.

**Fig. S5.** Complete frequency scan of the Brillouin spectral response measured using a lock-in amplifier, showing both the Brillouin gain and loss components. **a** Spectrum corresponding to a narrow Brillouin linewidth, typically observed at lower temperatures or in low-pressure gas conditions. **b** Spectrum corresponding to a broad Brillouin linewidth, characteristic of higher temperatures or denser gas environments. These measurements highlight how temperature and pressure influence acoustic damping in the gas medium.

**References**

1. Feynman, R. P. The Feynman lectures on physics. in vol. 1 Chapter 39-44 (Basic Books, New York, 2011).

2. Epstein, P. S. *Textbook of Thermodynamics*. (Andesite Press, 2017).

3. Bailyn, M. *A Survey of Thermodynamics*. (AIP Press, Woodbury, NY, 1994).

4. Hirschfelder, J. O., Curtiss, C. F. & Bird, R. B. *Molecular Theory of Gases and Liquids*. (Wiley, Hoboken, NJ, 2010).

5. Callen, H. B. *Thermodynamics and an Introduction to Thermostatistics*. vol. 2 (1980).

6. Stokes, G. G. On the Theories of the Internal Friction of Fluids in Motion, and of the Equilibrium and Motion of Elastic Solids. in *Mathematical and Physical Papers* vol. 1 75–129 (Cambridge University Press, Cambridge, 2009).

7. Bird, R. B., Stewart, W. E. & Lightfoot, E. N. *Transport Phenomena*. (J. Wiley, New York, 2002).

8. Chapman, S. & Cowling, T. G. *The Mathematical Theory of Non-Uniform Gases: An Account of the Kinetic Theory of Viscosity, Thermal Conduction and Diffusion in Gases*. (Cambridge Univ. Pr, Cambridge, 1999).

9. Pauli, W. & Enz, C. P. *Wave Mechanics*. (Dover Publications, Mineola, N.Y, 2000).

10. Backhurst, J. R., Harker, J. H., Richardson, J. F. & Coulson, J. M. Fluid Flow, Heat Transfer and Mass Transfer. in *Coulson & Richardson’s Chemical Engineering* vol. 1 (Elsevier, Amsterdam, 2010).

11. Eucken, A. Über das Wärmeleitvermögen, die spezifische Wärme und die innere Reibung der Gase. *Phys Z* **14**, 324–332 (1913).

12. Landau, L. D., Lifshit︠s︡, E. M. & Landau, L. D. *Fluid Mechanics*. (Pergamon Press, Oxford, England ; New York, 1987).

13. Huber, M. *et al.* NIST Reference Fluid Thermodynamic and Transport Properties Database (REFPROP) Version 10 - SRD 23. National Institute of Standards and Technology https://doi.org/10.18434/T4/1502528 (2018).

14. Yang, F., Gyger, F. & Thévenaz, L. Intense Brillouin amplification in gas using hollow-core waveguides. *Nat. Photonics* **14**, 700–708 (2020).

15. Rysselberghe, P. V. Remarks concerning the Clausius-Mossotti Law. *J. Phys. Chem.* **36**, 1152–1155 (1932).

16. Fellay, A. Extreme temperature sensing using Brillouin scattering in optical fibers. (PhD thesis, EPFL, 2003). doi:10.5075/epfl-thesis-2728.

17. Thévenaz, L., Fellay, A. & Scandale, W. Brillouin gain spectrum characterization in optical fibres  from 1 to 1000 K. in *16th International Conference on Optical Fiber Sensors* Technical Digest, Paper Tu2-2, 38-41 (IECE, Nara, Japan, 2003).

18. Ding, M. *et al.* Gas Flow Behaviour in Hollow Core Fibres under Cryogenic Temperature. in *2024 IEEE Photonics Society Summer Topicals Meeting Series (SUM)* 1–2 (IEEE, Bridgetown, Barbados, 2024). doi:10.1109/SUM60964.2024.10614527.

19. Gladstone, J. H. & Dale, T. P. XIV. Researches on the refraction, dispersion, and sensitiveness of liquids. *Philos. Trans. R. Soc. Lond.* **153**, 317–343 (1863).
